# Lipid profiles in the cerebrospinal fluid of rats with 6-hydroxydopamine-induced lesions as a model of Parkinson’s disease

**DOI:** 10.3389/fnagi.2022.1077738

**Published:** 2023-01-20

**Authors:** Jiewen Qiu, Guoyou Peng, Yuting Tang, Shiyin Li, Zengfu Liu, Jiayun Zheng, Yunxin Wang, Hanqun Liu, Lijian Wei, Yilin Su, Yuwan Lin, Wei Dai, Zhiling Zhang, Xiang Chen, Liuyan Ding, Wenyuan Guo, Xiaoqin Zhu, Pingyi Xu, Mingshu Mo

**Affiliations:** ^1^Department of Neurology, The First Affiliated Hospital of Guangzhou Medical University, Guangzhou, China; ^2^Department of Physiology, School of Basic Medical Sciences, Guangzhou Medical University, Guangzhou, China; ^3^Department of Internal Medicine, Huilai People’s Hospital, Jieyang, China

**Keywords:** lipid profiles, cerebrospinal fluid, 6-hydroxydopamine, Parkinson’s disease, biomarkers

## Abstract

**Background:**

Parkinson’s disease (PD) is a progressive neurodegenerative disease with characteristic pathological abnormalities, including the loss of dopaminergic (DA) neurons, a dopamine-depleted striatum, and microglial activation. Lipid accumulation exhibits a close relationship with these pathologies in PD.

**Methods:**

Here, 6-hydroxydopamine (6-OHDA) was used to construct a rat model of PD, and the lipid profile in cerebrospinal fluid (CSF) obtained from model rats was analyzed using lipidomic approaches.

**Results:**

Establishment of this PD model was confirmed by apomorphine-induced rotation behaviors, loss of DA neurons, depletion of dopamine in the striatum, and microglial activation after 6-OHDA-induced lesion generation. Unsupervised and supervised methods were employed for lipid analysis. A total of 172 lipid species were identified in CSF and subsequently classified into 18 lipid families. Lipid families, including eicosanoids, triglyceride (TG), cholesterol ester (CE), and free fatty acid (FFA), and 11 lipid species exhibited significantly altered profiles 2 weeks after 6-OHDA administration, and significant changes in eicosanoids, TG, CE, CAR, and three lipid species were noted 5 weeks after 6-OHDA administration. During the period of 6-OHDA-induced lesion formation, the lipid families and species showed concentration fluctuations related to the recovery of behavior and nigrostriatal abnormalities. Correlation analysis showed that the levels of eicosanoids, CE, TG families, and TG (16:0_20:0_18:1) exhibited positive relationships with apomorphine-induced rotation behaviors and negative relationships with tyrosine hydroxylase (TH) expression in the midbrain.

**Conclusion:**

These results revealed that non-progressive nigrostriatal degeneration induced by 6-OHDA promotes the expression of an impairment-related lipidomic signature in CSF, and the level of eicosanoids, CE, TG families, and TG (16:0_20:0_18:1) in CSF may reveal pathological changes in the midbrain after 6-OHDA insult.

## Introduction

Parkinson’s disease (PD) is characterized by pathological progressive degeneration involving massive loss of dopaminergic (DA) neurons in the substantia nigra (SN), dopamine-depleted striatum, microglial activation, and accumulation of α-synuclein (α-syn) (Balestrino and Schapira, 2020). Gene-environment interactions may contribute to the development of PD (Balestrino and Schapira, 2020). As an ideal environmental toxin, 6-hydroxydopamine (6-OHDA) is used to construct unilateral lesions in the SN of rats, cats, and primates (Deumens et al., 2002). The 6-OHDA-induced PD model recapitulates most pathological abnormalities except α-syn accumulation and alters dopamine-related behaviors, such as apomorphine-induced rotation (Deumens et al., 2002). However, 6-OHDA exposure represents a non-progressive insult, and the resulting pathological and behavioral abnormalities can be reversed by drugs, which differs from PD treatment responses observed in the clinic (Cicchetti et al., 2002; Deumens et al., 2002). The 6-OHDA model has been applied to explore sensitive biomarkers for nigrostriatal function.

Lipid and lipoprotein metabolism play essential roles in the occurrence and development of neurodegenerative disorders (Farmer et al., 2020). Apolipoprotein E, as a component of lipoprotein, may disrupt the blood-brain barrier (BBB) and increase the risk of Alzheimer’s disease (AD) (Fullerton et al., 2001; Chen et al., 2021). Some mutations in the glucosylceramidase-beta (GBA) gene encoding the lysosomal hydrolase glucocerebrosidase (GCase) involved in membrane lipid composition represent high risk factors for PD and dementia with Lewy bodies (Barkhuizen et al., 2016; Gan-Or et al., 2018). PD patients with GBA mutations exhibit dramatic changes in lipid concentrations in serum, including increased ceramide and sphingomyelin levels and decreased phosphatidic acid levels (Guedes et al., 2017). Lipid disorders are also closely related to 6-OHDA-related pathological abnormalities, such as the loss of DA neurons (Xicoy et al., 2019; Elabi et al., 2021). In lipid families, eicosanoids have special profiles in aging mice and AD patients (Valcarcel-Ares et al., 2019; Duro et al., 2022), and cholesterol ester (CE) can aggregate in triggering receptor expressed on myeloid cells 2 (TREM_2_) KO and AD-variant human iPSC microglia (García-Sanz et al., 2021; Magno et al., 2021). The dysregulated metabolism of triglyceride (TG) has been found in PD pathology (Sharma and Taliyan, 2018; Kao et al., 2019; Brekk et al., 2020; Toscano et al., 2020). Regarding lipid species, several anionic and zwitterionic lipids bind to TREM_2_ receptors in microglia involved in the recognition of abnormally deposited proteins, such as Aβ and α-syn, in neurodegenerative diseases (Wang et al., 2015). As a characteristic product of lipid peroxidation, 15-hydroperoxy-arachidonoyl-phosphatidylethanolamine can activate the ferroptotic death signal of DA neurons in PD (Sun et al., 2021; Wang et al., 2022). Regarding dopamine depletion, diets rich in saturated fat may change dopamine precursor availability related to dopamine concentrations in the midbrains of humans (Hartmann et al., 2020). Thus, lipid metabolism exhibits a close relationship with PD pathology and is considered to be an ideal biomarker for diagnosis.

In many studies on clinical PD, the search for biomarkers focuses on serum. Due to BBB blockade, biomarkers in cerebrospinal fluid (CSF) exhibit better accuracy and sensitivity to reflect the pathological changes of nigrostriatal in PD (Pan and Nicolazzo, 2018). Thus, this study aims to explore lipid metabolism in CSF obtained from rats with 6-OHDA lesions and evaluate the association between lipids in CSF and PD-related abnormalities.

## Materials and methods

### Animals

**FIGURE 1:**
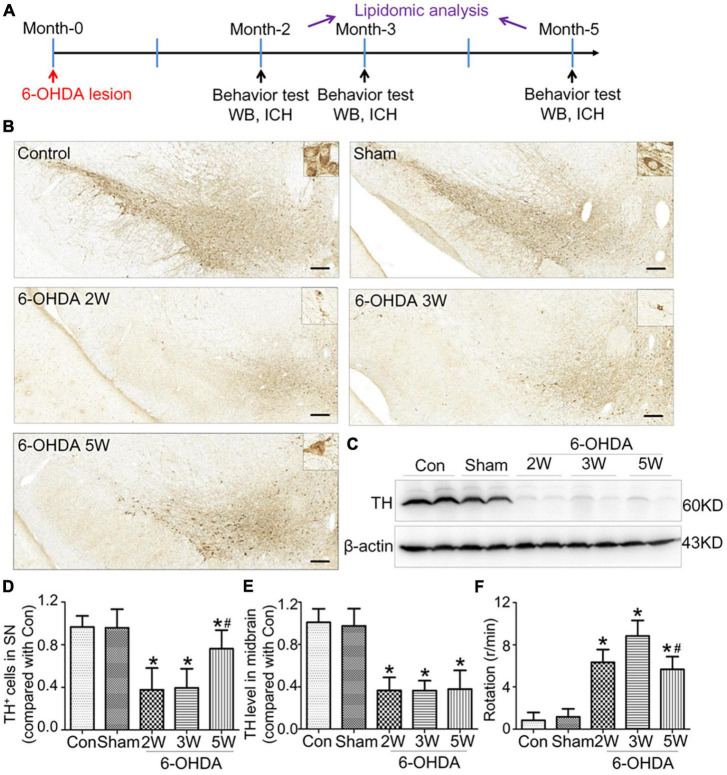
Non-progressive nigrostriatal degeneration in the 6-OHDA model. **(A)** Schematic diagram of experimental procedure. **(B)** SN sections showed TH immunoreactivity and nigrostriatal degeneration at 2, 3, and 5 weeks after unilateral lesion formation as assessed by immunohistochemistry staining. **(C)** Western blotting was used to detect the reduction in TH in the midbrain at different time points. The graph shows the number of TH^+^ cells in the SNpc as assessed by stereology in panel **(D)** and the TH level in the midbrain in Western blot in panel **(E)**. **(F)** The apomorphine-induced rotational response was evaluated by the number of anti-clockwise rotations performed over 30 min, and the mean number per minute was calculated. Data are represented as mean ± S.E.M. *P < 0.05 vs. Control, ^#^*P* < 0.05 vs. 2 weeks group. 6-OHDA, 6-hydroxydopamine; SN, substantia nigra; TH, tyrosine hydroxylase; SNpc, substantia nigra pars compacta. Scale bar: 50 μm.

Adult male Sprague-Dawley rats (4 months old, weighing 350–490 × g) were purchased from Jiangsu Aniphe Biolaboratory (Jiangsu, China) and were maintained under specific-pathogen-free conditions with free access to food and water and a 12-h light/12-h dark cycle in the Laboratory Animal Center (Guangzhou Medical University, Guangzhou, China). All procedures used in the present study were performed in accordance with the guidelines on animal care. All experiments were approved by the Animal Experiments and Ethics Committee of Guangzhou Medical University (No. 2020143, 21 August 2020). The rats were assigned randomly to five groups: the control group without injection, the sham group with injection of vehicle only, and the three 6-OHDA groups injected with 6-OHDA for observation times of 2, 3, and 5 weeks, separately. The experimental procedure was showed in Figure 1A.

### 6-OHDA lesions

The neurotoxic rat model of PD was constructed by a unilateral injection of 6-OHDA into the medial forebrain bundle (MFB) as reported previously (Mo et al., 2018). Using the rat brain atlas of Paxinos and Watson (2007) as a guide, 6-OHDA (Sigma-Aldrich, Shanghai, China) was injected into the MFB based on the following coordinates: anterior-posterior, −4.4 mm; medial-lateral, −1.2 mm; dorsal-ventral, −7.8 mm relative to bregma. The rats were placed in a stereotaxic device under 1% pentobarbital sodium anesthesia. Here, 6-OHDA was dissolved at 2 μg per 1 μL in vehicle solution that contained 0.2% ascorbic acid in an equal volume of 0.9% NaCl. In the PD rats, 4 μL of the 6-OHDA solution was administered at a rate of 400 nl/min using a Hamilton syringe equipped with a 30-gauge needle. The needle was left after injection for an additional 10 min and withdrawn at a speed of 1 mm/min. The same coordinates were used in rats undergoing sham operation, but only vehicle was delivered.

### Apomorphine-induced rotation test

To evaluate the presence of 6-OHDA lesions, the apomorphine-induced turning rate was assessed according to a previously described protocol (0.5 mg/kg, i.p. apomorphine) (Mo et al., 2018). At 2, 3, and 5 weeks after 6-OHDA injection, the rats were injected with 0.5 mg/kg apomorphine (Sigma-Aldrich, Shanghai, China) at 0.5 mg/ml dissolved in NaCl by intraperitoneal injection. Then, the rats were transferred individually to a transparent plastic cylinder, and the rotations were recorded for 30 min in a self-constructed rotameter according to a previously reported method (Mo et al., 2018; Palese et al., 2019). The total number of complete 360° anti-clockwise rotations was counted, and the mean number per minute was calculated and expressed in the Section “Results.”

### Immunohistochemical staining

Rats were anesthetized with chloral hydrate and then perfusion-fixed with precooled 4% paraformaldehyde (Sigma-Aldrich, Shanghai, China) followed by 0.9% NaCl. The brains were separated, postfixed in 4% formalin (PFA) for 24 h and cryoprotected in 30% w/v sucrose in phosphate buffer saline (PBS) for dehydration. After embedding in OCT medium (Tissue-Tek, Sakura Finetek, USA), the brains were sectioned at 15 or 20 μm using a freezing microtome. The brain sections were incubated with primary antibodies against tyrosine hydroxylase (TH, Millipore, MAB318, 1:400), glial fibrillary acidic protein (GFAP, Cell signaling Technology, 80788, 1:1,000), peroxisomal biogenesis factor 19 (Pex19, ABclonal, A5476, 1:50), and Iba1 (Proteintech, 10904, 1:200). For immunofluorescence staining, the sections were counterstained with 4’,6-diamidino-2-phenylindole (DAPI). The slices were canned by a digital pathology scanner (PRECICE 500B, UNIC Technologies Inc., Beijing, China) following the manufacturer’s instructions. Morphometric analyses were performed using the ImageJ program (NIH, Bethesda, MD, United States).

The number of TH^+^ neurons in the substantia nigra pars compacta (SNpc) was counted using an optical fractionator. Every three sections from a total of 9–12 sections per animal throughout the entire SNpc were counted in the lesion side under a × 20 objective view. The SNpc was identified by defined anatomic landmarks. TH^+^ cells with optimally visualized nuclei in one view were considered a valid count. To count the number of Iba1^+^ cells based on immunofluorescence staining, three different sections containing 1,200 μm × 1,400 μm standardized areas in the SNpc were selected randomly as described previously (Gramage et al., 2022). The total area of Iba1^+^ cells was calculated as the whole image fluorescence. After the mean background fluorescence was subtracted, the program ImageJ was used to identify and count the number of Iba1^+^ cells. The density of Iba1^+^ cells was determined based on the number of cells and area. Three sections were quantified per rat.

Quantification of TH expression in the striatum was performed using the Fiji image analysis system in ImageJ as previously described (Mo et al., 2018; Gramage et al., 2022). The whole striatum region in both hemispheres was identified as a region of interest. TH immunoreactivity was measured in both hemispheres. After the sections were scanned, the images were converted to binary mode. All the rats shared the same threshold. The percentage was considered as the TH intensity of the lesion side compared with the contralateral side in the same section. A total of 4–5 striatal sections were assessed per rat.

### Western blot

The midbrain was separated, and its proteins were extracted as previously reported (Mo et al., 2018; Gramage et al., 2022). Protein concentrations were estimated using a Nanodrop™ 8,000 Spectrophotometer (Thermo Fisher Scientific, Waltham, MA, USA). The protein extracts were run on a 4–12% sodium dodecyl sulfate polyacrylamide gel electrophoresis (SDS-PAGE) gel and then transferred to polyvinylidene fluoride (PVDF) membranes. The membranes were then incubated with primary antibodies against TH (Millipore, MAB318, 1:2000) or β-actin (Bioworld, AP0060, 1:5000). After washing with Tris-buffered saline containing 0.1% Tween-20, the membranes were incubated with HRP-conjugated secondary antibodies. An enhanced chemiluminescence system (ECL, Millipore) was used for visualization. The blots were assessed using the Image Lab Software 6.0 (Bio-Rad Laboratories, Hercules, CA, USA), and the results were normalized to the intensity of β-actin.

### UPLC-MS/MS analysis of CSF

After anesthesia with 1% pentobarbital sodium (40 mg/kg, i.p.), the rats were placed in a stereotaxic device. The head was position at a 135 degree angle to the body. After shaving and disinfection, a 2-cm longitudinal incision along the back midline in the occipital was generated to expose the foramen magnum. The insulin needle was used directly to puncture the dura mater to the cerebellomedullary cistern. Then, the CSF was drawn for UPLC-MS/MS analysis.

Lipid extraction from CSF was performed as previously reported (Nogueras et al., 2019). Fifty microliters of CSF sample was mixed with Bligh-Dyer extraction and internal lipid standards to 1 ml (Supplementary Data 1). After incubation in an ultrasonic water bath with a frequency of 40 kHz and a power of 100 W for 5 min, 500 μl Milli-Q water was added, and the sample was centrifuged at 12,000 r/min at 10°C. Then, 500 μl supernatant was collected and mixed with 100 μl mobile phase B. Then, 20 μl of each sample was added to a pool as a quality control. The detection system included a Shim-pack UFLC SHIMADZU CBM30A ultra-performance liquid chromatograph (UPLC) (SHIMADZU, JAPAN) coupled with a QTRAP5500 tandem mass spectrometer (MS/MS) (SCIEX, USA). Chromatographic separation was performed on a Thermo Accucore™ C30 column (2.1 mm × 100 mm, 2.6 μm). The column temperature was maintained at 45°C. Mobile Phases A (60/40 acetonitrile/water) and B (10/90 acetonitrile/water) both contained 0.1% acetic acid and 10 mmol/L ammonium formate. The injection volume was 2.0 μL, and the flow rate was 0.35 mL/min. The gradient elution program was set as follows: 20% B at the beginning; 30–60% B between 2–9 min, 85–90% B between 9–15.5 min, 95% B between 15.5–17.5 min; 20% B for 17.5–20 min. The desolvation temperature was set as 500°C. The MS voltage in positive and negative modes was set to 5,500 V and −4,500 V, respectively. The pressures of the curtain gas, gas II, and ion source gas were 35 psi, 55 psi, and 45 psi, respectively. The ion pairs were detected by optimized collision energy and declustering potential in the triple quadrupole UPLC-MS/MS (Supplementary Data 2).

### Lipidomic analysis in CSF

The UPLC-MS/MS analysis used a widely targeted metabolome method based on the Metware database (Metware Biotechnology Co., Ltd., Wuhan, Hubei, China) (Liu et al., 2023). The retention time, ion pair information, and secondary spectrum data were used for semi-quantitative analysis. The semi-quantitative analysis was performed by the multiple reaction monitoring modes (MRM) of triple quadrupole mass spectrometry, and the data were analyzed using Analyst 1.6.3 software (AB SCIEX, Framingham, MA, USA) and MultiQuant (version 3.0, AB SCIEX). For peak area determinations, an individual area of the same metabolite was normalized to the integrated area of all peaks. All sample extracts were mixed as the quality control (QC), and one QC sample was inserted into every ten samples. Total ion flow diagrams of various QC samples were used to evaluate the repeatability of metabolite extraction and detection. The lipid species were classified as eicosanoids, triglycerides (TGs), phosphatidylcholine (PC), sphingomyelin (SM), free fatty acids (FFAs), diglyceride (DG), lysophosphatidylcholine (LPC), phosphatidylethanolamine (PE), ceramide (Cer), carnitine (CAR), monoglyceride (MG), cholesterol ester (CE), phosphatidylserine (PS), hexosylceramide (HexCer), sphingomyelin (SPH), butyric acid (BA), and phosphatidylglycerol (PG).

Unsupervised and supervised methods were used to evaluate whether the lipidomic signature in CSF could be determined after the induction of lesions using 6-OHDA. In the comparison between the early stage after 6-OHDA treatment and the control, the principal component analysis (PCA) results showed that PC-1 and PC-2 accounted for 26.2 and 16.5%, respectively (Supplementary Data 3A). The partial least squares-discriminant analysis (PLS-DA) results indicate that 22% of the total variance can be explained by component-1, and 12% of the total variance can be explained by component-2 (Supplementary Data 3B). Using the orthogonal partial least-squares discrimination analysis (OPLS-DA) method, the orthogonal T score and T score were 22.4 and 9.3%, respectively (Supplementary Data 3C). OPLS-DA data were subject to S-plot analysis (Supplementary Data 3D). The Q (Deumens et al., 2002) intercept value of −0.237 in the permutation test (*n* = 200) suggested that the OPLS-DA model had statistically effective quality and robustness (Supplementary Data 3E). The lipids with the top 15 VIP values were identified and are shown in the VIP score plot (Supplementary Data 3F).

The same methods were selected to compare the control with late stage 6-OHDA lesions. The PCA results showed that PC-1 and PC-2 accounted for 23.8 and 14.9%, respectively (Supplementary Data 4A). The PLS-DA results showed that 15.1% of the total variance can be explained component-1 and 14.2% of the total variance can be explained by component-2 in the comparison of the late stage with the control (Supplementary Data 4B). Using the OPLS-DA method, the orthogonal T score, and T score were 22.1 and 6% (Supplementary Data 4C). The S-plot and permutation test were performed, and the Q (Deumens et al., 2002) intercept values were −0.301 (Supplementary Datas 4D, E). The lipids with the top 15 variable importance in projection (VIP) values are shown in the VIP score plot (Supplementary Data 4F).

To evaluate lipid peroxidation, the malondialdehyde (MDA) content in CSF was determined by a detection kit from Solarbio (Solarbio Science and Technology, Beijing, China) following the manufacturer’s instructions.

### Statistical analysis

The data are presented as the means ± standard errors of the means. Comparisons between two groups accepted the unpaired *t*-test or Mann-Whitney test after the normality test. Comparisons among multiple groups were analyzed by one-way ANOVA followed by the least significant difference (LSD) *post hoc* test. Data analyses were performed using statistical product and service solutions (SPSS) 16.0 (IBM, USA), and *p* < 0.05 indicated a statistically significant difference.

In lipidomic analysis, SIMCA version 15 software (Umetrics, Umeå, Sweden) and MetaboAnalyst software (Version 5.0^1^) were used for the PCA, PLS-DA, and OPLS-DA. The volcano plot, box plot, and heatmap were drawn using GraphPad Prism (GraphPad Prism^®^ Software version 8.0.2 for Windows; La Jolla, CA, United States) and Origin software (Version 2022, OriginLab Inc., USA). The metabolites with variable importance in the projection (VIP), false discovery rate (FDR) adjusted *p*-value, and absolute log_2_Fold change (FC) were used for cluster analysis with Origin software (Version 2022, OriginLab Inc., USA). Violin plots were generated using Hiplot.^2^ Pearson correlations were analyzed by Origin software (Version 2022, OriginLab Inc., USA). In the Pearson correlation and linear mixed-effects model, the significance of each variable was assessed at a level of 0.05.

## Results

### Non-progressive nigrostriatal degeneration in the 6-OHDA model of PD

**FIGURE 2:**
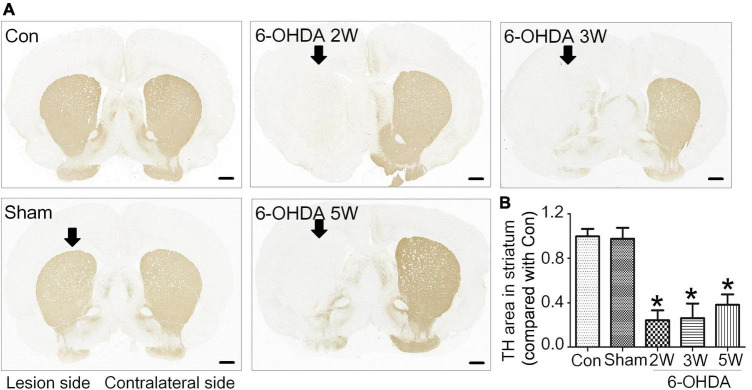
Non-progressive degeneration of the striatum in the 6-OHDA model. **(A)** Striatal sections showed TH immunoreactivity and nigrostriatal degeneration at 2, 3, and 5 weeks after unilateral lesion formation as assessed by immunohistochemical staining. **(B)** The graph shows the TH level in the midbrain. Data are represented as mean ± S.E.M. *P < 0.05 vs. Control. 6-OHDA, 6-hydroxydopamine; TH, tyrosine hydroxylase. Scale bar: 50 μm.

Rats with nigrostriatal degeneration induced by 6-OHDA, as an animal model of PD, were assessed using apomorphine before sacrifice. The timepoints of assessment were 2, 3, and 5 weeks after 6-OHDA administration. At these three time points, the number of DA neurons in the SN decreased significantly in PD rats compared with the sham group as assessed by IHC (fold change: 0.31, 0.33, and 0.64, respectively) and WB (fold change: 0.33, 0.34, and 0.38, respectively, Figures 1B–E), similar to the TH-positive area in the striatum by IHC (fold change: 0.16, 0.16, and 0.27, respectively, Figures 2A, B) and WB (fold change: 0.21, 0.22, and 0.31, respectively, Supplementary Data 5), and astrocyte density in SN (Supplementary Data 6). The number of TH^+^ cells in the locus coeruleus (LC) also decreased significantly at 2, 3, and 5 weeks after 6-OHDA injection with fold changes of 0.35, 0.37, and 0.45, respectively (Supplementary Data 7). These effects were accompanied by dramatically increased apomorphine-induced rotations with fold changes of 5.52, 6.23, and 3.96 (Figure 1F). The density of microglia in the SN increased 4.17-fold at 2 weeks and 3.81-fold at 3 weeks after 6-OHDA administration, as shown by immunofluorescence staining (Supplementary Data 8). Interestingly, at 5 weeks after lesion induction, apomorphine-induced rotations, the number of DA neurons, the density of microglia in the SN, and TH^+^ cells in the LC all markedly recovered compared to those noted 2 weeks after lesion induction, and the TH-positive area in the striatum also showed a recovery trend without significance. These data suggest that functional recovery or compensatory nigrostriatal impairment may occur in the 6-OHDA model, and this non-progressive characteristic may help to explore and evaluate ideal biomarkers of nigrostriatal lesions.

### Lipid families from CSF at different stages of 6-OHDA lesions

**FIGURE 3:**
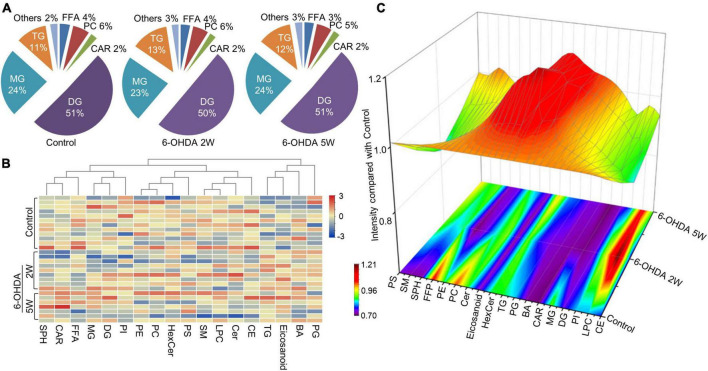
Lipid families in CSF after 6-OHDA-induced lesion formation. The detected lipid species were classified into 18 families. **(A)** The proportion of each lipid family in the control, 6-OHDA 2W and 5W groups is listed based on expression levels. **(B)** The heatmap representing the entire 18 lipid families obtained by UPLC-MS/MS. **(C)** Three-dimensional color graphs show the intensity trend of lipid families in CSF under normal conditions and in the different stages of 6-OHDA-induced lesion formation. The color scale reflects the molecular intensity compared with the control. 6-OHDA, 6-hydroxydopamine; TG, triglyceride; PC, phosphatidylcholine; SM, sphingomyelin; FFA, free fatty acid; DG, diglycerides; LPC, lysophosphatidylcholine; PE, phosphatidylethanolamine; CER, ceramide; CAR, carnitine; MG, Monoglyceride; CE, cholesterol ester; PS, phosphatidylserine; HexCer, hexosylceramide; SPH, sphingomyelin; BA, butyric acid; PG, phosphatidylglycerol.

**FIGURE 4:**
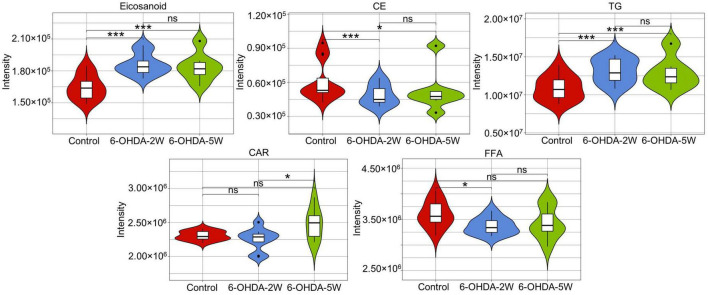
Characteristic lipid families in CSF after 6-OHDA-induced lesion formation. Violin plot showing the expression and distribution of lipid families with FDR adjusted *p*-value < 0.05 in the 6-OHDA 2W or 5W groups compared with control. Differences were determined by Student’s *t*-test, with *p < 0.05 and ***p < 0.001 determined by the Mann-Whitney test. 6-OHDA, 6-hydroxydopamine; CE, cholesterol ester; CAR, carnitine; FFA, free fatty acid; TG, triglyceride. ns, not significant at 0.05 probability level.

The lipidomic signature in CSF may be a considerable biomarker that is sensitive to nigrostriatal degeneration in PD (Hwangbo et al., 2022). Based on the results of TH expression and rotation tests, abnormal performances are observed at an early stage approximately 2 weeks after 6-OHDA lesion. At this time point, the global lipidomic differences in CSF were detected and analyzed. A total of 172 lipid species were found and classified into 18 families. The lipid families included 2 eicosanoids, 9 FFAs, 6 Bas, 1 PS, 1 PI, 1 SPH, 1 HexCer, 1 PG, 8 CARs, 5 CEs, 2 CERs, 21 DGs, 4 LPCs, 6 MGs, 29 PCs, 10 PEs, 14 SMs, and 51 TGs. According to the percentage of expression, the DG, MG, TG, and PC families accounted for 51, 24, 11, and 6% in the control group, 50, 23, 13, and 6% in the 6-OHDA 2W group, 51, 24, 12, and 5% in the 6-OHDA 5W group, respectively (Figure 3A). The overview of lipid families was provided by hierarchical clustering analysis (Figure 3B). During the period of 6-OHDA lesion formation, six lipid families showed upregulated trends, whereas 12 lipid families exhibited downregulated trends at the early stage of 2 weeks. Among them, eicosanoids, CE, TG, and FFA families had significantly different expression levels. Over the next 3 weeks, CAR levels increased significantly. At the late stage of 5 weeks, CE still had a significantly lower level, eicosanoids, TG and CAR exhibited a partial recovery, but their levels remained higher than those of the control without significance, and FFA almost recovered to normal levels (Figure 3C). The eicosanoid, CE, TG, and FFA families exhibited 1. 13-, 0. 81-, 1. 21-, and 0.93-fold changes with significance in expression in the 6-OHDA 2W group, and 1. 11-, 0. 87-, 1. 14-, and 0.95-fold changes in the 6-OHDA 5W group compared with the control, respectively, CAR exhibited a 1.84-fold change between two 6-OHDA groups (Figure 4 and Supplementary Data 9).

### Lipid species from CSF at the different stages of 6-OHDA lesions

**FIGURE 5:**
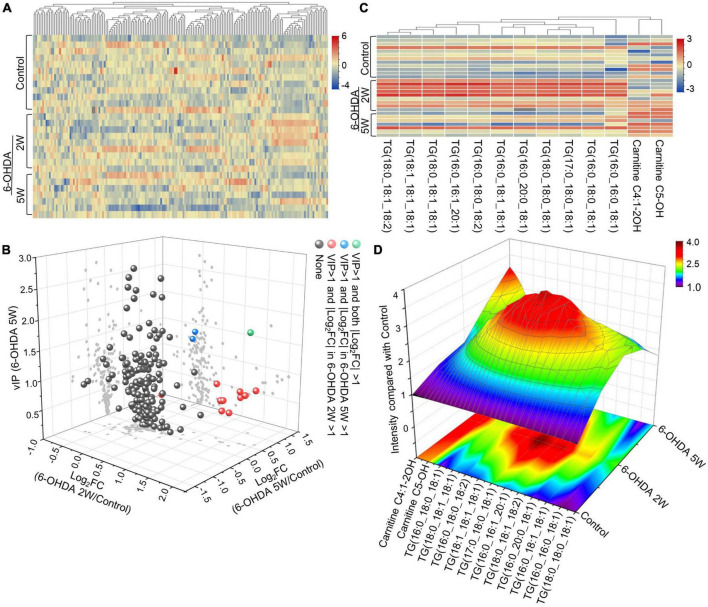
Lipid signature in CSF after 6-OHDA-induced lesion formation. **(A)** Heatmap representing all 172 lipid species obtained by UPLC-MS/MS. **(B)** Three-dimensional volcano plot showing that 13 lipid species with |Log_2_FC| > 1 and VIP > 1 in the early stage of 6-OHDA-induced lesion formation exhibited different changing trends in the subsequent 3-week period. **(C)** The 13 lipid species were used to generate the lipidomic signature as shown in the heatmap. **(D)** Three-dimensional color graphs show the intensity trend of 13 lipid species in CSF under normal conditions and in the different stages of 6-OHDA-induced lesion formation. The color scale reflects the molecular intensity compared with the control. 6-OHDA, 6-hydroxydopamine; VIP, variable importance in the projection; FC, fold change.

**FIGURE 6:**
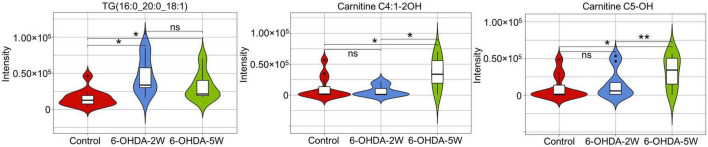
Characteristic lipid species in CSF after 6-OHDA-induced lesion formation. Violin plot showed the expression and distribution of lipid species with FDR adjusted *p*-value < 0.05 in the 6-OHDA 5W compared with control groups. Differenced determined by Student’s *t*-test, being *p < 0.05 and **p < 0.01 by Mann–Whitney test. 6-OHDA, 6-hydroxydopamine; TG, triglyceride. ns, not significant at 0.05 probability level.

A complete overview of 172 lipid species was provided by hierarchical clustering (Figure 5A and Supplementary Data 10). At the early stage of 2 weeks, 11 lipid species were upregulated. Over the next 3 weeks, 10 of the 11 species almost recovered to normal concentrations. Only TG (16:0_20:0_18:1) maintained an increased level compared with the control, and the new lipids carnitine C5-OH and carnitine C4:1-2OH were significantly upregulated at the late stage of 5 weeks (Figures 5B, C). Above 13 lipid species also met the selection criteria of FDR adjusted *p*-value < 0.05 in 2W or 5W 6-OHDA groups compared with the control, and were used to draw the characterized lipidomic signature for PD (Figure 5C). The 13 lipid species exhibited different changing trends during the period of 6-OHDA lesion formation (Figure 5D). Among them, TG(16:0_16:0_18:1), TG(16:0_18:0_18:1), TG(17:0_18:0_18:1), TG(18:0_18:0_18:1), TG(16:0_20:0_18:1), TG(16:0_18:1_18:1), TG(16:0_18:0_18:2), TG(16:0_16:1_20:1), TG(18:0_18:1_18:2), TG(18:1_18:1_18:1), and TG(18:0_18:1_18:2) exhibited 2. 22-, 3. 38-, 3. 40-, 3. 64-, 3. 00-, 2. 83-, 3. 19-, 2. 73-, 4. 00-, 2. 45-, and 2.79-fold changes with significance in expression in the 6-OHDA 2W group compared with the control, respectively. After a of 3-week period of recovery, the level of 169 lipid species had no significant change except TG(16:0_20:0_18:1), carnitine C5-OH, and carnitine C4:1-2OH with 2. 08-, 3. 02-, and 3.19-fold changes in the 6-OHDA 5W group (Figure 6 and Table 1).

**TABLE 1:** Lipid species in CSF after 6-OHDA-induced lesion formation.

Lipids	6-OHDA 2W/control		6-OHDA 5W/control		6-OHDA 5W/2W	
	Fold change	Adj.P	Fold change	Adj.P	Fold change	Adj.P
Carnitine C5-OH	1.52	0.82	3.02	0.02	3.02	0.03
Carnitine C4:1-2OH	1.51	0.82	3.19	0.02	3.19	0.01
TG(16:0_16:0_18:1)	2.22	0.04	1.11	0.94	1.11	0.03
TG(16:0_18:0_18:1)	3.38	0.03	1.41	0.91	1.41	0.04
TG(17:0_18:0_18:1)	3.40	0.04	1.42	0.91	1.42	0.06
TG(18:0_18:0_18:1)	3.64	0.03	1.42	0.91	1.42	0.03
TG(16:0_20:0_18:1)	3.00	0.03	2.08	0.03	2.08	0.78
TG(16:0_18:1_18:1)	2.83	0.03	1.27	0.91	1.27	0.04
TG(16:0_18:0_18:2)	3.19	0.03	1.22	0.94	1.22	0.02
TG(16:0_16:1_20:1)	2.73	0.03	1.27	0.91	1.27	0.04
TG(18:0_18:1_18:2)	4.00	0.03	1.55	0.91	1.55	0.04
TG(18:1_18:1_18:1)	2.45	0.03	1.36	0.91	1.36	0.82
TG(18:0_18:1_18:2)	2.79	0.03	1.27	0.91	1.27	0.50

Some studies have shown that 6-OHDA-induced lipid peroxidation is related to dopaminergic dysfunction (Lee et al., 2009; Seet et al., 2010). We explored lipid peroxidation in the CSF and detected the expression of Pex19 in the SN. The MDA content showed an increasing trend at 2 weeks after 6-OHDA lesion without a significant difference (Supplementary Data 11A). Pex19 also had no significant difference in expression in the SN at 2 and 5 weeks after 6-OHDA lesion (Supplementary Data 11B).

### Lipid metabolism in CSF follows the recovery of nigrostriatal impairment

**FIGURE 7:**
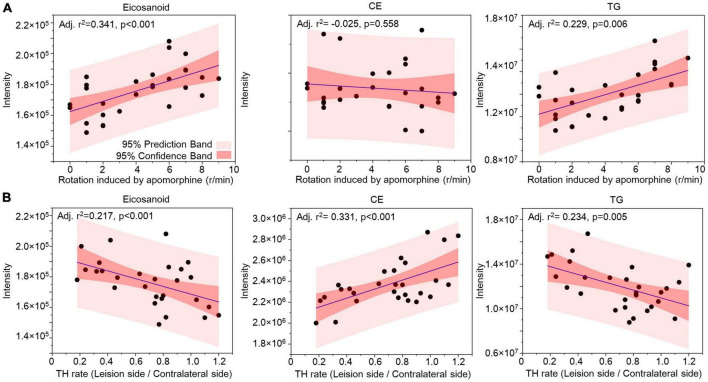
Relationship analysis between lipid families in CSF and 6-OHDA-induced lesions. The correlations between eicosanoids, CE, and TG in CSF with the rotation induced by apomorphine **(A)** and TH rate **(B)** were analyzed by Pearson correlation. The 95% prediction band and 95% confidence band are marked with light red and dark red areas, respectively. CE, cholesterol ester; TG, triglyceride; TH, tyrosine hydroxylase.

**FIGURE 8:**
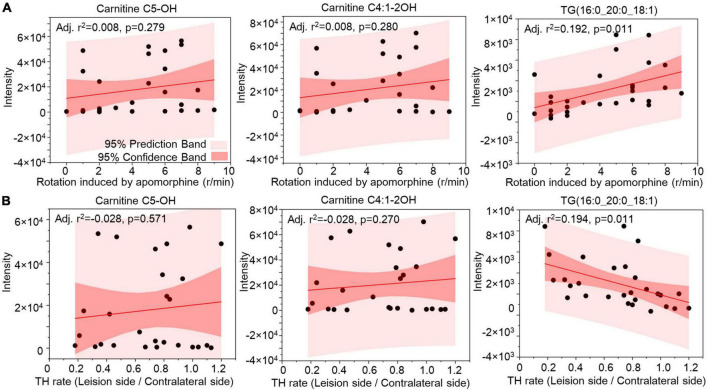
Relationship analysis between lipid species in CSF and 6-OHDA-induced lesions. The correlations between Carnitine C5-OH, Carnitine C4:1-2OH, and TG(16:0_20:0_18:1) in CSF with the rotation induced by apomorphine **(A)** and TH rate **(B)** were analyzed by Pearson correlation. The 95% prediction band and 95% confidence band were marked with light red and dark red areas, respectively. TG, triglyceride; TH, tyrosine hydroxylase.

Correlation analysis was used to evaluate the lipids in CSF as biomarkers for nigrostriatal degeneration in the 6-OHDA model. The rotation induced by apomorphine, as a behavior detection, can help to estimate the lesion of 6-OHDA in SN. Eicosanoids, CE, and TG were selected by their changed expression at 5 weeks after 6-OHDA lesion. The levels of eicosanoids and TG had a positive relationship with rotation behavior with *P*-values of <0.001 and 0.006, respectively (Figure 7A). The negative relationship of eicosanoids and TG with TH expression in the SN also exhibited different levels of significance with *P*-values of <0.001 and 0.005, respectively (Figure 7B). CE exhibited a positive relationship with TH expression with a *P*-value < 0.001 but an unclear relationship with rotation behavior with a *P*-value of 0.558. Regarding lipid species, carnitine C5-OH, carnitine C4:1-2OH, and TG (16:0_20:0_18:1) were also selected based on late-stage changes in expression. All of the lipid species exhibited significant positive relationships with rotation behavior, including carnitine C5-OH with a *P*-value of 0.279, carnitine C4:1-2OH with a *P*-value of 0.280, and TG (16:0_20:0_18:1) with a *P*-value of 0.011 (Figure 8A). TG (16:0_20:0_18:1) exhibited a negative relationship with TH expression in the SN with a *P*-value of 0.011. Both carnitine C5-OH and C4:1-2OH had generally positive relationships with TH expression (Figure 8B). These associations suggested that the eicosanoids, TG families, and TG (16:0_20:0_18:1) may be sensitive biomarkers of nigrostriatal degeneration in PD.

## Discussion

Clinical and preclinical diagnosis remains a major challenge for PD. When PD patients exhibit motor symptoms, up to 50–70% of DA neurons in the SN are lost (Orth and Tabrizi, 2003). The CSF lipidomic imbalance has been found in postmortem PD patients (Fernández-Irigoyen et al., 2021). Our study aims to explore biomarkers of PD that help realize early diagnosis and intervention for disease. Here, we used lipidomic approaches to develop a special lipid signature expressed in 6-OHDA-induced PD rats and found changes in the expression of lipid families and species in CSF that followed the progression of 6-OHDA lesions. We suggest that eicosanoids, CE, TG, and TG (16:0_20:0_18:1), which are related to 6-OHDA-induced pathological and behavioral abnormalities, may represent suitable biomarkers for PD.

The lipid families in CSF were classified into eicosanoids, FFA, BA, PS, PI, SPH, HexCer, PG, CAR, CE, CER, DG, LPC, MG, PC, PE, SM, and TG in this study. Among them, eicosanoids, DG, and TG were significantly upregulated compared with the control. Many studies have investigated to the role of TG in serum associated with ischemic cerebrovascular disease, but few studies have focused on the function of TG in neurodegenerative diseases (Shamim et al., 2018). In non-demented aging persons, TG levels in serum are inversely correlated with executive function (Parthasarathy et al., 2014). Some studies suggest that increased serum TG exhibits a close relationship with poor cognition (He et al., 2016), whereas other studies report either no relation (Henderson et al., 2003) or suggest the opposite conclusion (Lv et al., 2019). Circulating TG can increase the activity of the reward circuit in brain function (Cansell et al., 2014) and may be related to smaller cortical thickness in older persons with cerebral Aβ burden (Llado-Saz et al., 2015). TG was proposed to cross the BBB and induce special central receptor resistance, including leptin and insulin, which resulted in functional changes in feeding and cognition (Banks et al., 2004, 2018). TG metabolism also affects DA release, which plays multiple pivotal roles in reward systems, associative learning, motor planning, and decision-making (Ramos-Lopez et al., 2018; Berland et al., 2019, 2020). In PD patients, the concentration of TG in the SN is correlated with inflammation-attenuating signaling (Brekk et al., 2020). TG in the diet can affect the function of DA neurons to modulate dopamine-dependent behaviors and promote microglial polarization in the SN (Cansell et al., 2014; Toscano et al., 2020). Some studies hold the opinion that a high-fat diet includes neurotoxins and accelerates the loss of DA neurons in PD (Sharma and Taliyan, 2018; Kao et al., 2019). Regarding TG in CSF, relative reports on PD are rare. This study showed that the TG family and TG (16:0_20:0_18:1) exhibited significantly upregulated levels at 2 weeks after 6-OHDA-induced lesion formation. These upregulated levels partly recovered in the subsequent 3 weeks but were still higher than those noted in the control at 5 weeks. The pathological and behavioral abnormalities in the 6-OHDA model were most serious at 2 weeks, and their severity was consistent with the change in TG levels in the CSF. The above evidence indicated that the levels of the TG family members and TG (16:0_20:0_18:1) in CSF may be indicative of the presence of SN lesions in PD.

Eicosanoids and FFA are other lipid families that exhibited significantly changed expression in CSF at 2 weeks after 6-OHDA-induced lesion formation in this study. Eicosanoids, as amphipathic molecules, are derived from the oxidation of arachidonic acid and polyunsaturated fatty acids (Tapiero et al., 2002). Its subfamilies include prostaglandins, leukotrienes, thromboxanes, resolvins, lipoxins, protectins, isoprostanes, maresins, endocannabinoids, and others (Tapiero et al., 2002). Eicosanoids in the central nervous system regulate neuronal functions, including memory and learning, cerebral blood flow, sleep, and neuroinflammation (Biringer, 2019; Regulska et al., 2021; Duro et al., 2022). In aging mice, altered eicosanoid profiles in the hippocampus exhibit a close relationship with inflammation, synaptic dysfunction, and cognitive impairment (Valcarcel-Ares et al., 2019). The eicosanoid lipidome was activated in the brains of AD patients and was especially increased in carriers of the APOE4 allele (Duro et al., 2022). Regarding PD, relevant reports are lacking. This PD study showed that significantly upregulated levels at both 2 and 5 weeks after 6-OHDA-induced lesion formation were found in the eicosanoid family but not in its lipids. The changed expression of the eicosanoid family potentially included all the small differences in its lipids to achieve significance. This finding suggests that the eicosanoid family may be more suitable as a biomarker for PD than its lipids.

Regarding FFA, its accumulation in the brain is associated with some inherited neurological diseases, including Refsum disease and X-linked adrenoleukodystrophy (Schonfeld and Reiser, 2016). In AD patients, lower FFA levels were noted in apolipoprotein E4 carriers than in non-carriers (Duro et al., 2022). FFA may prevent the assembly of amyloid and tau filaments (Kruska and Reiser, 2011; Barracchia et al., 2020). FFAs, including palmitic acid, oleic acid, and stearic acid, have all been shown to have low concentrations in serum from PD patients in the preclinical stage (Havelund et al., 2017; Gonzalez-Riano et al., 2021), and were suggested as potential biomarkers of PD in a follow-up study (Havelund et al., 2017; Konjevod et al., 2022). Alpha-linolenic acid, classified as a polyunsaturated fatty acid, can inhibit 6-OHDA-induced dopaminergic neurodegeneration in worms (Shashikumar et al., 2015). However, the roles of eicosanoids and FFA in nigrostriatal function and the pathogenesis of PD remain unclear. In this study, significantly upregulated FFA levels were exclusively noted 2 weeks after 6-OHDA-induced lesion formation but not at 5 weeks. This result suggested that FFAs in CSF may be released by damaged DA neurons or other cells, which were present in limited numbers at 5 weeks. Thus, alterations in FFA expression in CSF may serve as a useful biomarker that helps to identify pathological changes in the early stage of PD. More details regarding the function and expression of eicosanoids and FFA in PD are still needed.

Cholesterol ester is known as a storage form of cholesterol and contributes to cholesterol transport in the brain (Jin et al., 2019; Gliozzi et al., 2021). In neurodegenerative diseases with abnormal protein aggregates, TREM2 as a kind of lipid-sensor in microglia, is a genetic susceptibility factor for AD (Nugent et al., 2020). CE aggregates can be found in TREM2 KO and AD-variant human iPSC microglia, and may be related to α-syn deposits (García-Sanz et al., 2021; Magno et al., 2021). Abnormal lysosomal cholesterol accumulation was found in isolated fibroblasts from PD patients carrying the N370S GBA1 mutation (García-Sanz et al., 2017). The concentration of 24-hydroxycholesterol esters also decreased in serum from PD (Havelund et al., 2017; Di et al., 2018). Another study showed that triacylglycerol, but not cholesterol, was selected as the main serum lipid used to distinguish PD patients from leucine-rich repeat kinase 2 (LRRK2) mutation carriers (Galper et al., 2022). The relationship between CE and PD risk is still unclear. Here, we showed that 6-OHDA-induced lesions decreased the level of the CE family in the CSF in both early and late stages. This suggests that cholesterol disorder may play an important role in PD pathology. More details of CE functions and mechanisms in PD also need further study.

Carnitine C5-OH and carnitine C4:1-2OH exhibited a special expression profile in which upregulated levels were found only at 5 weeks after 6-OHDA-induced lesion formation. Acetyl-L-carnitine has been a research hotspot in AD (Pennisi et al., 2020). Some studies have suggested that acetyl-L-carnitine can protect against inflammatory processes, revert the neurodegeneration caused by excitotoxicity, and play a preventive role in AD (Bodaghi-Namileh et al., 2018; Kepka et al., 2020; Pennisi et al., 2020). In a clinical study, pathway enrichment analysis suggested that the carnitine shuttle was an important pathway related to drug naïve PD patients (Sinclair et al., 2021). The 12–14 long-chain acylcarnitines with low levels in serum at the early stage of PD have a high specificity and moderate sensitivity for disease diagnosis (Saiki et al., 2017). In 6-OHDA-induced rat and chronic 1-Methyl-4-phenyl-1,2,3,6-tetrahydropyridine (MPTP)-induced mouse models, acetyl-l-carnitine can protect the immunoreactivity of TH and DTA in the nigral-striatal system, and slow PD progression (Afshin-Majd et al., 2017; Burks et al., 2019). The role of acetyl-L-carnitine in PD remains unclear. We found upregulated acetyl-L-carnitine expression at the late stage of 6-OHDA-induced lesion formation with improved pathological and behavioral abnormalities in a rat model. This finding suggested that carnitine C5-OH and carnitine C4:1-2OH may be related to the recovery of nigrostriatal lesions. It seems that different lipid profiles were found at different stages of 6-OHDA-induced lesion formation. A single lipid family or species is not sufficient to depict the complete picture of 6-OHDA-induced lesion formation in the brain. Different expression patterns involving multiple lipid families and species exhibit more sensitivity and accuracy for evaluating PD progression.

The products of lipid oxidation are considered ideal biomarkers for PD diagnosis in the clinic. As their members, hydroxyeicosatetraenoic acid and cholesterol oxidation products were found to have elevated concentrations in urine from PD patients (Lee et al., 2009). Other lipids, such as F2-isoprostanes and cholesterol oxidation products, both showed similarly increased concentrations in serum from PD (Lee et al., 2009; Seet et al., 2010). Compared with F2-isoprostanes, isofurans had more favored formation under oxygen tension (Fessel et al., 2003). A study found that isofurans, but not F2- isoprostane, had an upregulated trend in the SN of patients with PD and dementia with Lewy body (DLB) (Fessel et al., 2003). This suggests that the products of lipid oxidation may have special metabolic characteristics in different body fluids and tissues in PD patients. In this study, we found that lipid oxidation in the CSF and Pex19 in the SN did not change dramatically at 2 and 5 weeks after 6-OHDA-induced lesions. We inferred that lipid peroxidation products induced by 6-OHDA may have been metabolized after 2 weeks. Further studies are still needed to confirm this possibility.

Various neurotoxic and genetic animal models have been applied for PD studies. The classic transgenic models have difficulty reproducing the complete features of PD in the clinic, for example, α-Syn, Parkin, and DJ-1 knockout mice (Chia et al., 2020). The 6-OHDA model has advantages of PD-like unilateral behavioral phenotypes and predictable degeneration in DA neurons (Chia et al., 2020). *In vitro*, a 6-OHDA-treated SH-SY5Y cell model showed a series of lipid changes found in the brains of PD patients and animal models (Xicoy et al., 2020). The cholesterol levels in serum also showed a significantly reduced trend in 6-OHDA-treated rabbits (Meara et al., 1992). 6-OHDA was suggested as a good promoter of ferritin-dependent lipid peroxidation (Monteiro and Winterbourn, 1989). Here, we showed a special lipid profile in CSF from a PD rat model. This finding suggests that 6-OHDA is suitable as a neurotoxin to construct a PD model for exploring lipid metabolism.

Our study has some limitations. Our data are not strong enough to disclose the direct relationship between the lipid profile after 6-OHDA-induced lesions and neurodegeneration in PD rats. Additional studies will be needed to confirm this relationship and disclose the underlying mechanisms.

## Conclusion

In conclusion, 6-OHDA-induced lesions cause changes in the lipidomic profile of CSF, and these changes exhibit potential value as a potential diagnostic tool for PD. Characterization of lipid metabolism profiles that are altered following 6-OHDA-induced lesion formation, especially eicosanoids, CE, TG families, and TG (16:0_20:0_18:1), may open a new window for the evaluation of nigrostriatal impairment in PD and help to develop new anti-PD drugs.

## Data availability statement

The original contributions presented in this study are included in the article/Supplementary material, further inquiries can be directed to the corresponding authors.

## Ethics statement

The animal study was reviewed and approved by Animal Experiments and Ethics Committee of Guangzhou Medical University (No. 2020143, 21 August 2020).

## Author contributions

MM, XZ, and PX conceived and designed the study and interpreted the experiments. YT, LW, YS, and JQ performed the study and prepared the initial draft of the manuscript. MM, PX, GP, and YT supervised the project. WD, JQ, YL, SL, ZL, JZ, YW, WG, ZZ, LD, HL, and XC provided the experimental samples and data. All authors read and approved the final submission.

## Footnotes

1
https://www.metaboanalyst.ca/

2
https://hiplot.com.cn
