# Corrigendum: Lipid profiles in the cerebrospinal fluid of rats with 6-hydroxydopamine-induced lesions as a model of Parkinson's disease

**DOI:** 10.3389/fnagi.2024.1506373

**Published:** 2024-10-31

**Authors:** Jiewen Qiu, Guoyou Peng, Yuting Tang, Shiyin Li, Zengfu Liu, Jiayun Zheng, Yunxin Wang, Hanqun Liu, Lijian Wei, Yilin Su, Yuwan Lin, Wei Dai, Zhiling Zhang, Xiang Chen, Liuyan Ding, Wenyuan Guo, Xiaoqin Zhu, Pingyi Xu, Mingshu Mo

**Affiliations:** ^1^Department of Neurology, The First Affiliated Hospital of Guangzhou Medical University, Guangzhou, China; ^2^Department of Physiology, School of Basic Medical Sciences, Guangzhou Medical University, Guangzhou, China; ^3^Department of Internal Medicine, Huilai People's Hospital, Jieyang, China

**Keywords:** lipid profiles, cerebrospinal fluid, 6-hydroxydopamine, Parkinson's disease, biomarkers

In the published article, there was an error in the Funding statement. The funding number in “the General Project of Basic and Applied Basic Research of Guangzhou Bureau of Science and Technology (2060206)” was incorrect.

The correct Funding statement appears below.

