# Supplementary material for: Lipid profiles in the cerebrospinal fluid of rats with 6-hydroxydopamine-induced lesions as a model of Parkinson’s disease
Source: Front Aging Neurosci. 2023 Jan 20;14:1077738. doi: 10.3389/fnagi.2022.1077738 (PMC9895836; doi:10.3389/fnagi.2022.1077738)

**Supplemental data 1.** Internal standards for UPLC‒MS/MS

| Compounds | CAS | Company | Catalogue Number | Concentration(μM) |
| --- | --- | --- | --- | --- |
| LPC(12:0) | 20559-18-6 | Avanti | 855475P | 0.2 |
| LPE(14:0) | 123060-40-2 | Avanti | 856735P | 0.2 |
| Cer(d18:1/4:0) | 74713-58-9 | Avanti | 860504P | 0.2 |
| MG(17:0) | 5638-14-2 | Sigma-Aldrich | SMB00506 | 0.2 |
| PG(12:0/12:0) | 322647-27-8 | Avanti | 840435P | 0.2 |
| PC(13:0/13:0) | 71242-28-9 | Avanti | 850340P | 0.2 |
| PE(12:0/12:0) | 59752-57-7 | Avanti | 850702P | 0.2 |
| DG(12:0/12:0) | 60562-15-4 | Avanti | 800812P | 0.2 |
| TG(12:0/12:0/12:0) | 538-24-9 | Sigma-Aldrich | T4891 | 0.2 |
| TG(17:0/17:0/17:0) | 2438-40-6 | Sigma-Aldrich | T2151 | 0.2 |
| CE(17:0) | 24365-37-5 | Rhawn | R090798-1g | 2 |
| DG(17:0/17:0) | 98896-81-2 | Cayman | 26942 | 0.2 |
| FFA(16:0)-d31 | 39756-30-4 | sigma | 68277-25MG | 0.2 |

**Supplemental data 2.** Collision energy for UPLC‒MS/MS

| Compounds | Class | Collision energy(V) |
| --- | --- | --- |
| 3-Hydroxy-dodecanoyl-carnitine | CAR | 40 |
| PC(O-18:3/20:3) | PC-O | 40 |
| PC(O-20:2/24:3) | PC-O | 40 |
| PC(14:0/18:2) | PC | 40 |
| PC(O-18:3/20:2) | PC-O | 40 |
| Cer(d18:1/24:1) | Cer | 40 |
| CE(22:6) | CE | 20 |
| CE(20:4) | CE | 20 |
| SM(d18:1/20:1) | SM | 30 |
| SM(d18:2/14:0) | SM | 30 |
| SM(d18:0/16:0) | SM | 30 |
| SM(d18:2/24:1) | SM | 30 |
| 3-Hydroxy-dodecanoyl-carnitine | CAR | 40 |

**Supplemental data 3.** Multivariate statistics on the lipidomic signature at the early stage of 6-OHDA-induced lesion formation

(A) Nonsupervised analysis by PCA. (B) Supervised analysis by PLS-DA. (C) Supervised analysis by OPLS-DA. (D) The S-plot generated from the OPLS-DA model. (E) The permutation test of the OPLS-DA model. (F) VIP score plot and identified lipid species representing the top 15 values. Abbreviations: 6-OHDA, 6-hydroxydopamine; PCA, principal component analysis; PLS-DA, partial least discriminate analysis; OPLS-DA, orthogonal partial least discriminate analysis; VIP, variable importance in projection.


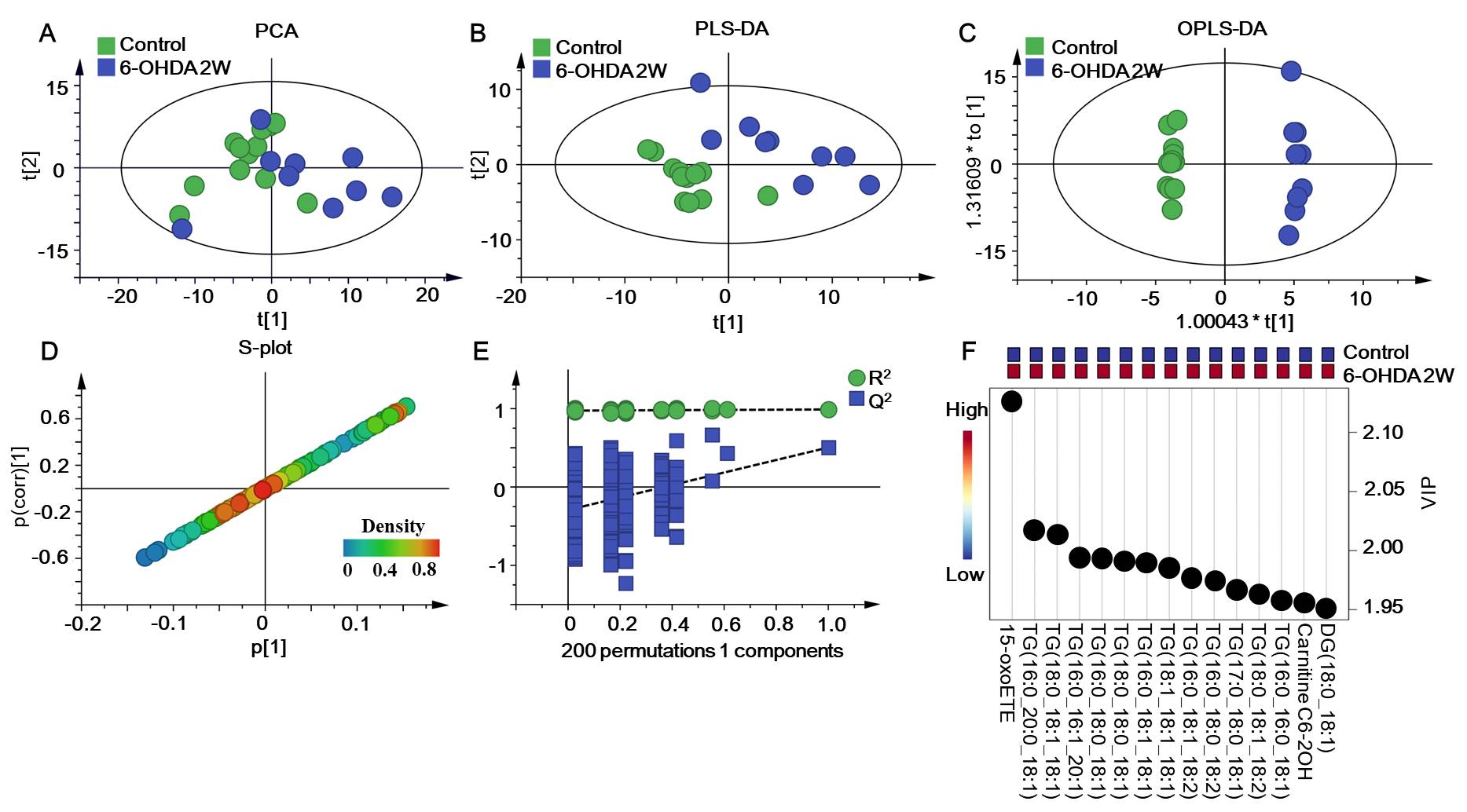


**Supplemental data 4.** Multivariate statistics on the lipidomic signature at the late stage of 6-OHDA-induced lesion formation

(A) Nonsupervised analysis by PCA. (B) Supervised analysis by PLS-DA. (C) Supervised analysis by OPLS-DA. (D) The S-plot generated from the OPLS-DA model. (E) The permutation test of the OPLS-DA model. (F) VIP score plot and identified lipid species representing the top 15 values. Abbreviations: 6-OHDA, 6-hydroxydopamine; PCA, principal component analysis; PLS-DA, partial least discriminate analysis; OPLS-DA, orthogonal partial least discriminate analysis; VIP, variable importance in projection.


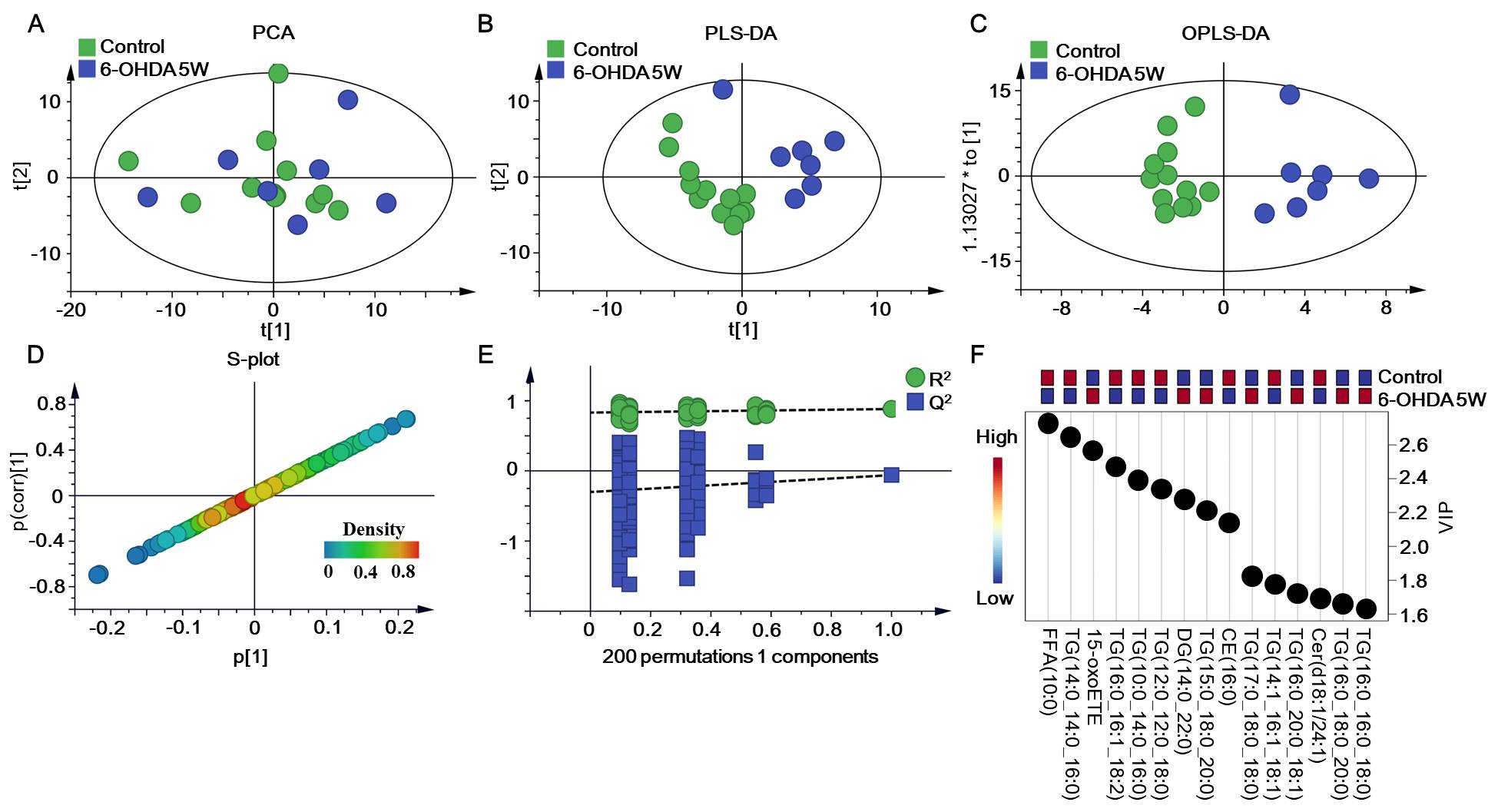


**Supplemental data 5** Degeneration of striatum in the 6-OHDA model

(A) Western blotting was used to detect the expression of TH in the striatum at different time points. (B) Histogram represented western blotting analysis. Data are represented as mean ± S.E.M. *P < 0.05 vs. Control. Abbreviations: 6-OHDA, 6-hydroxydopamine; TH, tyrosine hydroxylase.

**
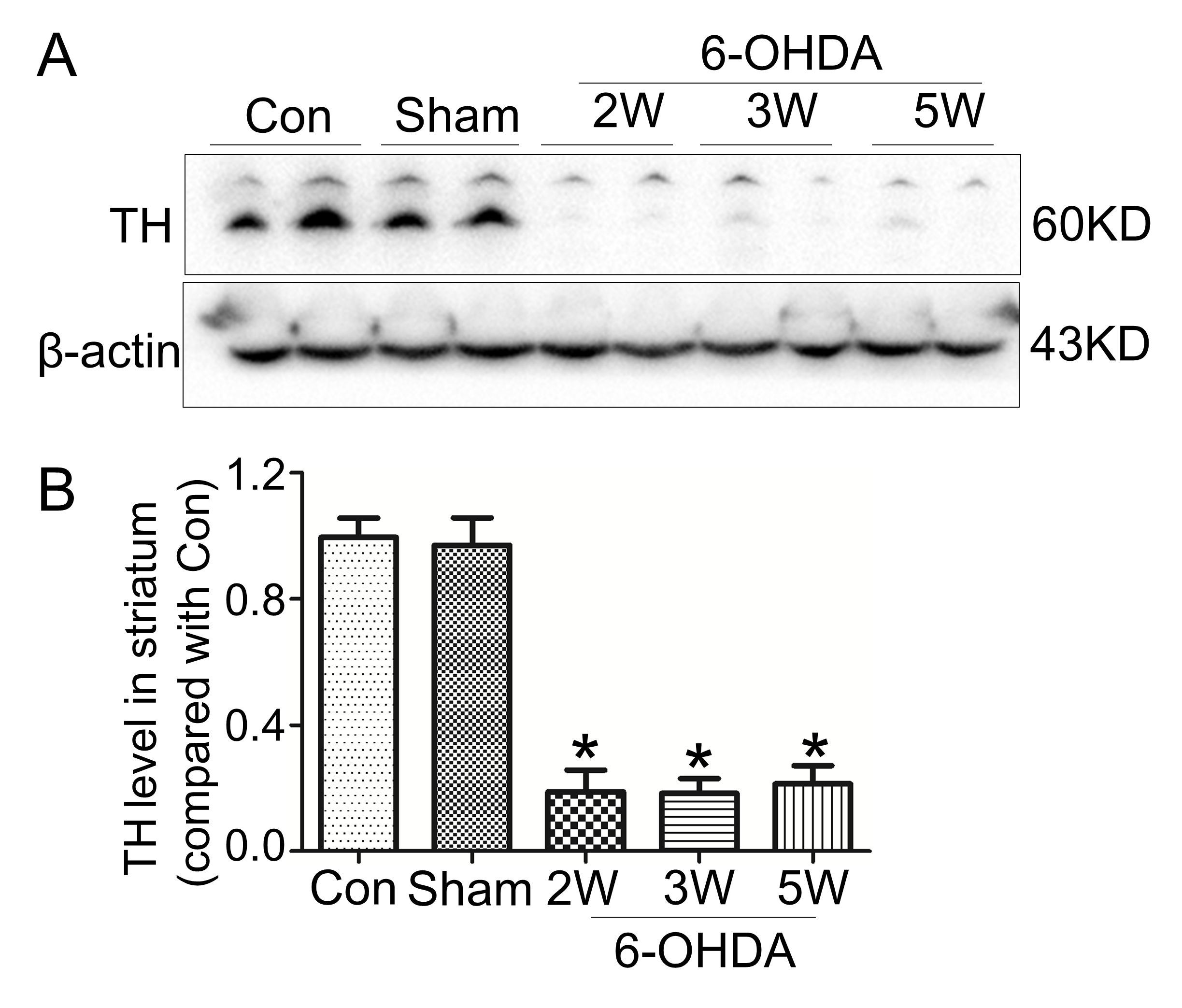
**

**Supplemental data 6** Astrocyte morphology in the SN of 6-OHDA rats

(A) Striatal sections showed the density and morphology of astrocyte in the SN at 2, 3 and 5 weeks after unilateral lesion formation as assessed by immunofluorescence staining. (B) The graph shows the astrocyte density in the SN. Data are represented as mean ± S.E.M. *P < 0.05 vs. Control. Abbreviations: 6-OHDA, 6-hydroxydopamine; SN, substantia nigra. Scale bar: 50 µm.

**
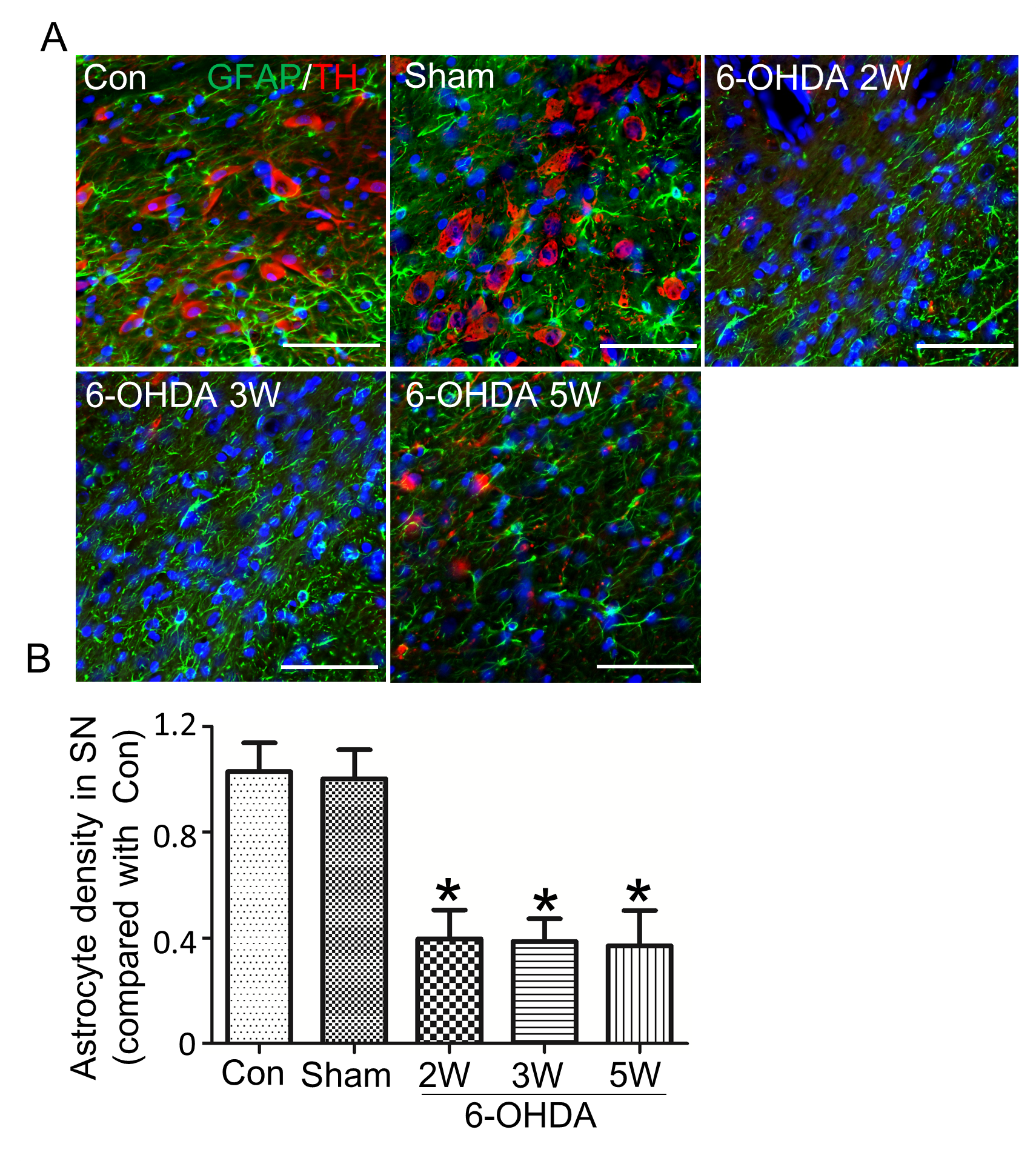
**

**Supplemental data 7** Impairment of the locus coeruleus in the 6-OHDA model

(A) Striatal sections showed TH immunoreactivity in LC at 2, 3 and 5 weeks after unilateral lesion formation as assessed by immunofluorescence. (B) The graph shows the number of TH^+^ cells in the LC. Data are represented as mean ± S.E.M. *P < 0.05 vs. Control. Abbreviations: 6-OHDA, 6-hydroxydopamine; TH, tyrosine hydroxylase; LC, locus coeruleus. Scale bar: 50 µm.

**
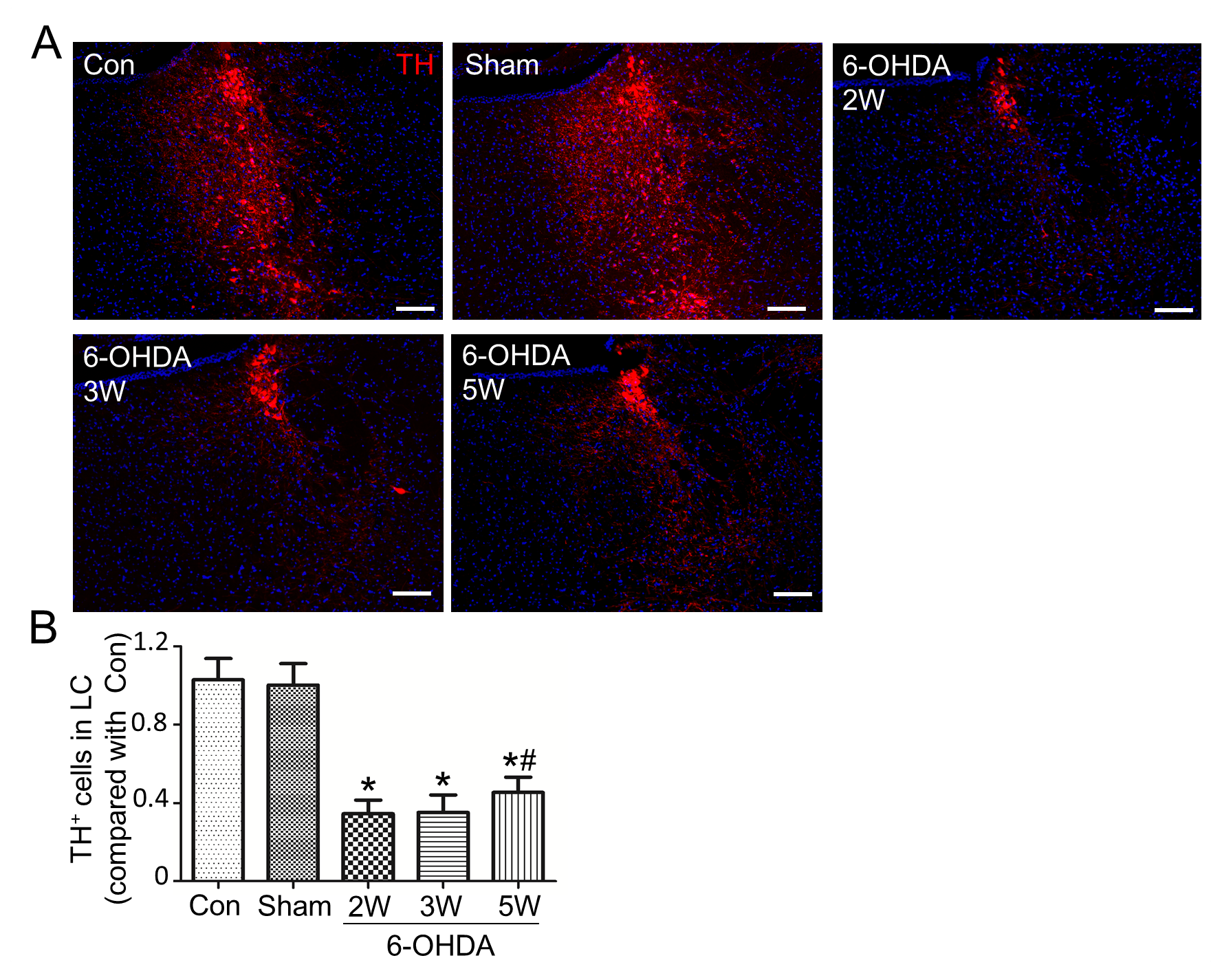
**

**Supplemental data 8.** Noncontinuous activation of microglia in the SN of 6-OHDA rats

(A) Striatal sections showed the density and morphology of microglia in the SN at 2, 3 and 5 weeks after unilateral lesion formation as assessed by immunofluorescence staining. (B) The graph shows the microglial density in the SN. Data are represented as mean ± S.E.M. *P < 0.05 vs. Control, ^#^P < 0.05 vs. 2W group. Abbreviations: 6-OHDA, 6-hydroxydopamine; SN, substantia nigra. Scale bar: 50 µm.

**Supplemental data 9.** Expression of lipid families from CSF

| Lipid family | Control | | 6-OHDA 2W | | | 6-OHDA 5W | | | adj.P  (5W v.s. 2W) |
| --- | --- | --- | --- | --- | --- | --- | --- | --- | --- |
|  | Mean | SE | Mean | SE | adj.P | Mean | SE | adj.P |  |
| BA | 641974.70 | 4789.50 | 675949.23 | 5460.01 | 0.191 | 647330.91 | 8892.65 | 0.860 | 0.353 |
| Eicosanoid | 164578.42 | 913.25 | 186036.00 | 1068.88 | 0.000 | 183499.86 | 1774.71 | 0.005 | 0.673 |
| FFA | 3600022.17 | 20533.58 | 3368226.89 | 16913.26 | 0.029 | 3420170.71 | 41598.70 | 0.193 | 0.673 |
| LPC | 144229.43 | 6727.87 | 129567.69 | 12607.77 | 0.746 | 163529.40 | 12042.83 | 0.646 | 0.545 |
| PC | 5911779.45 | 130781.80 | 5554795.96 | 161793.10 | 0.619 | 5378346.12 | 239626.08 | 0.519 | 0.836 |
| PI | 4685.98 | 201.16 | 4242.63 | 204.03 | 0.667 | 3292.86 | 132.80 | 0.184 | 0.262 |
| PS | 3749.81 | 90.33 | 3042.92 | 129.00 | 0.188 | 3827.49 | 230.55 | 0.907 | 0.308 |
| CAR | 2392079.74 | 16598.89 | 2253444.60 | 17034.85 | 0.115 | 2481523.77 | 31266.45 | 0.109 | 0.038 |
| CE | 60341.59 | 1381.94 | 48809.68 | 960.94 | 0.006 | 52437.97 | 2464.24 | 0.033 | 0.616 |
| SPH | 454249.17 | 10700.26 | 400634.44 | 13546.94 | 0.369 | 517051.43 | 20667.28 | 0.366 | 0.125 |
| Cer | 14143.08 | 265.38 | 14585.98 | 519.96 | 0.809 | 11367.84 | 382.29 | 0.084 | 0.151 |
| DG | 51056764.85 | 280688.73 | 50659689.60 | 510313.63 | 0.830 | 52513834.54 | 612768.43 | 0.448 | 0.453 |
| MG | 24218628.83 | 84515.16 | 23559716.56 | 139636.64 | 0.222 | 24613725.71 | 137142.93 | 0.440 | 0.107 |
| HexCer | 4767.21 | 123.63 | 5459.10 | 167.07 | 0.330 | 5108.60 | 299.74 | 0.700 | 0.721 |
| PE | 461347.13 | 4319.93 | 432219.88 | 5222.78 | 0.223 | 443939.13 | 7197.86 | 0.509 | 0.661 |
| PG | 3211.00 | 57.51 | 3571.26 | 32.45 | 0.178 | 3288.70 | 75.15 | 0.811 | 0.223 |
| SM | 627320.63 | 11036.97 | 551722.18 | 15931.66 | 0.249 | 540322.21 | 25055.97 | 0.264 | 0.895 |
| TG | 10876025.45 | 128934.45 | 13192662.47 | 170226.46 | 0.004 | 12446777.06 | 282570.42 | 0.009 | 0.457 |

**Supplemental data 10.** Expression of lipid species from CSF

| Lipids | Control | | 6-OHDA 2W | | | 6-OHDA 5W | | | adj.P  (5W v.s. 2W) |
| --- | --- | --- | --- | --- | --- | --- | --- | --- | --- |
|  | Mean | SE | Mean | SE | adj.P | Mean | SE | adj.P |  |
| taurolithocholic acid-3-sulfate | 9607.00 | 150.62 | 9349.34 | 130.01 | 0.92 | 9086.20 | 170.07 | 0.91 | 0.97 |
| 6,7-diketolithocholic acid | 517644.17 | 4167.82 | 549213.33 | 5468.12 | 0.65 | 531460.00 | 8263.54 | 0.94 | 0.90 |
| Ursocholic acid | 22333.00 | 339.01 | 23997.00 | 305.37 | 0.82 | 20700.86 | 338.01 | 0.91 | 0.43 |
| Glycocholic acid | 51578.00 | 1000.50 | 58779.33 | 1386.87 | 0.70 | 57143.43 | 1725.22 | 0.91 | 0.97 |
| Taurochenodeoxycholic acid | 26123.87 | 1804.03 | 21303.56 | 1182.14 | 0.88 | 15317.29 | 972.64 | 0.91 | 0.80 |
| Taurocholic acid | 14688.67 | 315.24 | 13306.67 | 199.17 | 0.82 | 13623.14 | 200.31 | 0.91 | 0.97 |
| 15-oxoETE | 115620.50 | 902.78 | 134418.89 | 724.71 | 0.03 | 130380.00 | 1101.76 | 0.39 | 0.82 |
| TxB3 | 48957.92 | 635.29 | 51617.11 | 704.31 | 0.82 | 53119.86 | 1803.93 | 0.91 | 0.97 |
| FFA(10:0) | 825968.33 | 7175.09 | 779856.67 | 8830.70 | 0.75 | 705548.57 | 5811.60 | 0.39 | 0.46 |
| FFA(14:0) | 151383.33 | 2465.12 | 142058.89 | 1843.09 | 0.82 | 150441.43 | 3400.56 | 0.98 | 0.87 |
| FFA(15:0) | 100509.75 | 1351.73 | 101633.44 | 834.72 | 0.94 | 103864.00 | 2674.30 | 0.94 | 0.97 |
| FFA(16:0) | 1019281.67 | 8159.90 | 983513.33 | 6265.81 | 0.82 | 1019352.86 | 9732.60 | 1.00 | 0.82 |
| FFA(17:0) | 52374.08 | 595.49 | 49549.00 | 523.03 | 0.82 | 52760.14 | 851.33 | 0.98 | 0.82 |
| FFA(18:0) | 1354316.67 | 12230.62 | 1237144.44 | 6963.56 | 0.26 | 1314271.43 | 26892.17 | 0.94 | 0.82 |
| FFA(18:1) | 43776.83 | 2916.16 | 32170.67 | 486.22 | 0.82 | 33297.00 | 972.09 | 0.91 | 0.97 |
| FFA(18:2) | 24385.00 | 2859.74 | 14982.78 | 485.64 | 0.82 | 12621.86 | 269.91 | 0.91 | 0.78 |
| FFA(22:3) | 28026.50 | 447.49 | 27317.67 | 469.13 | 0.92 | 28013.43 | 613.51 | 1.00 | 0.97 |
| LPC(18:0/0:0) | 6938.75 | 319.75 | 5935.42 | 439.84 | 0.88 | 5530.84 | 335.75 | 0.91 | 0.99 |
| PC(14:0_18:1) | 213040.83 | 1255.50 | 201444.44 | 848.62 | 0.33 | 204891.43 | 2028.57 | 0.91 | 0.91 |
| PC(18:1_18:1) | 11194.85 | 271.90 | 11509.46 | 363.38 | 0.94 | 12165.27 | 595.35 | 0.94 | 0.97 |
| PC(18:0_18:2) | 7677.54 | 455.70 | 7227.42 | 530.15 | 0.94 | 5811.55 | 572.99 | 0.91 | 0.91 |
| PC(18:0_20:4) | 13642.48 | 622.47 | 13070.82 | 972.52 | 0.94 | 12429.26 | 558.14 | 0.94 | 0.99 |
| PI(18:1_18:1) | 4685.98 | 201.16 | 4242.63 | 204.03 | 0.92 | 3292.86 | 132.80 | 0.91 | 0.82 |
| PS(18:0_18:0) | 3749.81 | 90.33 | 3042.92 | 129.00 | 0.65 | 3827.49 | 230.55 | 0.98 | 0.82 |
| Carnitine C2:0 | 5969.56 | 472.83 | 4242.72 | 238.08 | 0.82 | 5097.87 | 271.82 | 0.94 | 0.87 |
| Carnitine C5:0 | 88773.33 | 5802.39 | 79840.44 | 4637.53 | 0.92 | 142703.00 | 11391.71 | 0.91 | 0.50 |
| Carnitine C5-OH | 10494.63 | 1334.22 | 15958.36 | 2126.30 | 0.82 | 31744.02 | 2936.24 | 0.02 | 0.03 |
| Carnitine C6-2OH | 14171.75 | 113.51 | 12405.56 | 107.39 | 0.06 | 13933.57 | 285.70 | 0.97 | 0.50 |
| Carnitine C12-OH | 7868.19 | 957.49 | 5146.91 | 342.41 | 0.87 | 4119.84 | 274.62 | 0.91 | 0.87 |
| Carnitine C12:1-2OH | 25883.25 | 184.32 | 26254.44 | 273.57 | 0.92 | 26059.57 | 334.65 | 0.98 | 0.99 |
| Carnitine C4:1-2OH | 11440.90 | 1513.81 | 17318.39 | 2363.90 | 0.82 | 36523.04 | 3404.69 | 0.02 | 0.01 |
| Carnitine C5:1-2OH | 2213483.33 | 8228.25 | 2092277.78 | 11510.20 | 0.13 | 2221342.86 | 21809.02 | 0.98 | 0.50 |
| CE(16:0) | 6204.17 | 125.55 | 6010.97 | 165.77 | 0.93 | 4469.71 | 222.33 | 0.53 | 0.50 |
| CE(16:1) | 6782.20 | 109.73 | 5822.26 | 181.56 | 0.65 | 5907.70 | 51.06 | 0.91 | 0.99 |
| CE(18:1) | 11111.39 | 174.20 | 10958.21 | 190.75 | 0.94 | 11899.99 | 439.56 | 0.91 | 0.87 |
| CE(18:2) | 13498.42 | 231.35 | 11252.39 | 239.24 | 0.36 | 12375.54 | 526.44 | 0.91 | 0.87 |
| CE(20:4) | 22745.42 | 991.44 | 14765.86 | 584.80 | 0.43 | 17785.03 | 1634.19 | 0.91 | 0.89 |
| SPH(d16:1) | 454249.17 | 10700.26 | 400634.44 | 13546.94 | 0.82 | 517051.43 | 20667.28 | 0.91 | 0.61 |
| Cer(d18:1/24:0) | 10118.82 | 184.19 | 10736.99 | 338.08 | 0.90 | 8588.86 | 282.98 | 0.91 | 0.67 |
| Cer(d18:1/24:1) | 4024.26 | 112.95 | 3848.99 | 205.02 | 0.94 | 2778.99 | 201.82 | 0.91 | 0.81 |
| DG(8:0_16:0) | 51452.92 | 658.06 | 47076.78 | 594.55 | 0.65 | 47244.86 | 1393.67 | 0.91 | 0.99 |
| DG(12:0_14:0) | 7322.61 | 160.21 | 7046.76 | 181.59 | 0.92 | 6255.67 | 278.78 | 0.91 | 0.87 |
| DG(14:0_16:0) | 33502.92 | 283.76 | 32594.89 | 394.95 | 0.88 | 33280.57 | 498.23 | 0.98 | 0.97 |
| DG(15:0_16:0) | 7016.02 | 94.40 | 7207.09 | 138.08 | 0.92 | 7196.30 | 119.80 | 0.96 | 0.99 |
| DG(16:0_16:0) | 10571350.00 | 71281.79 | 10510955.56 | 119315.75 | 0.94 | 11151457.14 | 204421.03 | 0.91 | 0.86 |
| DG(14:0_18:0) | 50617.17 | 400.68 | 49699.33 | 501.25 | 0.92 | 52846.43 | 1117.14 | 0.91 | 0.86 |
| DG(12:0_20:0) | 56701.83 | 374.70 | 57037.56 | 684.74 | 0.94 | 59563.29 | 1222.82 | 0.91 | 0.89 |
| DG(17:0_16:0) | 32695.42 | 293.30 | 31346.00 | 447.41 | 0.82 | 32978.29 | 494.35 | 0.98 | 0.87 |
| DG(16:0_18:0) | 21130083.33 | 135837.66 | 21107444.44 | 219851.33 | 0.99 | 21502714.29 | 257427.14 | 0.94 | 0.97 |
| DG(14:0_20:0) | 91529.42 | 760.39 | 92517.56 | 1173.82 | 0.94 | 96627.57 | 721.13 | 0.91 | 0.87 |
| DG(17:0_18:0) | 62496.50 | 576.37 | 60454.44 | 834.05 | 0.88 | 59958.43 | 822.21 | 0.91 | 0.99 |
| DG(18:0_18:0) | 18492416.67 | 93076.73 | 18176222.22 | 204211.45 | 0.92 | 18969857.14 | 174982.29 | 0.91 | 0.87 |
| DG(16:0_20:0) | 140606.67 | 972.90 | 132490.11 | 2222.96 | 0.82 | 143588.57 | 2119.28 | 0.94 | 0.82 |
| DG(14:0_22:0) | 87332.58 | 659.08 | 90058.89 | 1385.83 | 0.88 | 98004.57 | 1234.37 | 0.39 | 0.76 |
| DG(19:0_18:0) | 28739.00 | 342.40 | 26335.56 | 396.98 | 0.66 | 31470.14 | 715.09 | 0.91 | 0.43 |
| DG(20:0_18:0) | 112508.00 | 836.36 | 113902.11 | 1416.40 | 0.93 | 120854.29 | 1549.05 | 0.91 | 0.82 |
| DG(14:0_16:1) | 54044.58 | 2618.32 | 66908.56 | 4759.35 | 0.82 | 55642.29 | 3355.62 | 0.98 | 0.91 |
| DG(16:0_16:1) | 11847.70 | 152.46 | 11968.21 | 142.17 | 0.94 | 10958.29 | 255.49 | 0.91 | 0.79 |
| DG(18:0_18:1) | 17127.58 | 149.01 | 21960.33 | 489.69 | 0.05 | 17676.14 | 286.78 | 0.94 | 0.43 |
| DG(16:1_18:1) | 6672.88 | 143.85 | 6752.34 | 156.98 | 0.94 | 6864.59 | 367.47 | 0.98 | 0.99 |
| DG(18:1_18:1) | 10701.07 | 426.69 | 9710.87 | 478.51 | 0.92 | 8795.70 | 229.19 | 0.91 | 0.95 |
| MG(14:0) | 61764.92 | 336.40 | 58731.00 | 435.28 | 0.53 | 60918.43 | 741.76 | 0.94 | 0.87 |
| MG(16:0) | 8542133.33 | 35977.62 | 8247033.33 | 40769.01 | 0.55 | 8612728.57 | 69533.25 | 0.97 | 0.62 |
| MG(17:0) | 35266.92 | 213.21 | 33088.56 | 322.57 | 0.46 | 34063.57 | 262.89 | 0.91 | 0.87 |
| MG(18:0) | 15499250.00 | 56370.85 | 15147333.33 | 101053.90 | 0.82 | 15827571.43 | 77605.45 | 0.91 | 0.61 |
| MG(20:0) | 61997.17 | 478.79 | 56038.00 | 325.70 | 0.11 | 59525.14 | 376.04 | 0.91 | 0.43 |
| MG(18:1) | 18216.50 | 132.33 | 17492.33 | 304.11 | 0.83 | 18918.57 | 361.90 | 0.91 | 0.86 |
| HexCer(d18:1/24:1) | 4767.21 | 123.63 | 5459.10 | 167.07 | 0.82 | 5108.60 | 299.74 | 0.94 | 0.97 |
| LPC(0:0/16:0) | 55285.83 | 2336.84 | 55362.22 | 5374.59 | 1.00 | 72950.86 | 5939.85 | 0.91 | 0.87 |
| LPC(16:0/0:0) | 65800.67 | 3362.28 | 54006.11 | 5573.12 | 0.88 | 71892.43 | 5155.42 | 0.97 | 0.87 |
| LPC(0:0/18:0) | 16204.18 | 817.99 | 14263.93 | 1290.43 | 0.92 | 13155.27 | 966.84 | 0.91 | 0.99 |
| PC(14:0_14:0) | 37959.17 | 220.09 | 39497.44 | 449.77 | 0.82 | 39867.86 | 562.00 | 0.91 | 0.99 |
| PC(16:0_14:0) | 20048.67 | 395.85 | 17699.78 | 565.64 | 0.82 | 18038.96 | 1043.98 | 0.91 | 0.99 |
| PC(15:0_16:0) | 6639.88 | 268.43 | 7723.98 | 454.11 | 0.87 | 6503.50 | 595.10 | 0.98 | 0.92 |
| PC(16:0_16:0) | 416975.00 | 9629.05 | 385764.44 | 12994.71 | 0.88 | 374020.00 | 16690.09 | 0.91 | 0.99 |
| PC(16:0_18:0) | 73653.92 | 1910.96 | 63613.00 | 2579.22 | 0.82 | 65707.14 | 2847.34 | 0.91 | 0.99 |
| PC(16:0_16:1) | 69193.42 | 1508.00 | 69490.89 | 2242.42 | 0.99 | 67570.86 | 3540.71 | 0.98 | 0.99 |
| PC(16:0_18:1) | 3372900.00 | 79019.89 | 3299933.33 | 91131.93 | 0.94 | 3156971.43 | 147306.93 | 0.94 | 0.97 |
| PC(17:0_18:1) | 24843.08 | 714.73 | 27471.67 | 1181.18 | 0.88 | 23050.14 | 1213.82 | 0.94 | 0.87 |
| PC(18:0_18:1) | 345251.67 | 7913.64 | 324102.22 | 10620.63 | 0.91 | 306430.00 | 16727.04 | 0.91 | 0.97 |
| PC(16:0_18:2) | 269065.00 | 14198.93 | 198344.89 | 13582.97 | 0.82 | 197075.00 | 12063.58 | 0.91 | 0.99 |
| PC(16:0_18:3) | 21696.50 | 414.29 | 22318.56 | 341.81 | 0.92 | 21206.14 | 720.10 | 0.98 | 0.95 |
| PC(16:0_20:3) | 16033.30 | 540.73 | 16527.34 | 767.91 | 0.94 | 15345.30 | 1284.83 | 0.98 | 0.97 |
| PC(16:0_20:4) | 434531.67 | 14300.13 | 359465.56 | 15827.72 | 0.82 | 357735.71 | 16941.73 | 0.91 | 0.99 |
| PC(18:0_22:4) | 11982.61 | 207.17 | 10207.34 | 355.72 | 0.65 | 11556.49 | 639.97 | 0.98 | 0.89 |
| PC(16:0_22:5) | 52235.17 | 2111.98 | 39340.00 | 2653.75 | 0.81 | 35864.86 | 1723.14 | 0.91 | 0.97 |
| PC(16:0_22:6) | 218911.67 | 4430.68 | 176187.78 | 4469.81 | 0.36 | 187861.43 | 9937.53 | 0.91 | 0.97 |
| PC(18:0_22:6) | 72565.75 | 2019.96 | 58918.67 | 2328.77 | 0.69 | 62191.57 | 3571.81 | 0.91 | 0.97 |
| PC(18:1_22:6) | 16374.06 | 560.47 | 14856.89 | 441.29 | 0.88 | 14476.57 | 1008.64 | 0.94 | 0.99 |
| PC(O-16:0_16:0) | 10628.12 | 299.76 | 11671.81 | 384.17 | 0.87 | 10360.76 | 489.85 | 0.98 | 0.87 |
| PC(O-18:1_16:0) | 54770.83 | 1114.31 | 60008.56 | 1536.11 | 0.82 | 56690.57 | 2970.52 | 0.98 | 0.97 |
| PC(O-18:0_18:2) | 14565.33 | 273.11 | 15092.44 | 289.77 | 0.92 | 13904.31 | 548.40 | 0.94 | 0.87 |
| PC(O-16:0_20:4) | 33866.42 | 691.15 | 37135.67 | 832.74 | 0.82 | 35473.14 | 1660.72 | 0.96 | 0.97 |
| PC(O-16:1_20:4) | 32268.92 | 670.61 | 29114.11 | 1065.07 | 0.82 | 30025.57 | 1626.06 | 0.94 | 0.99 |
| PC(O-18:1_20:4) | 18326.78 | 576.59 | 19463.56 | 514.53 | 0.92 | 17488.84 | 819.69 | 0.98 | 0.87 |
| PC(O-18:2_20:4) | 20936.83 | 501.83 | 17593.89 | 371.52 | 0.65 | 17632.46 | 1027.33 | 0.91 | 0.99 |
| PE(16:0_18:1) | 7563.03 | 197.00 | 7425.71 | 213.30 | 0.94 | 6259.30 | 182.08 | 0.91 | 0.77 |
| PE(20:1_16:0) | 3528.14 | 76.51 | 3641.36 | 166.06 | 0.94 | 2974.36 | 193.54 | 0.91 | 0.87 |
| PE(18:1_18:1) | 20151.67 | 512.41 | 17887.11 | 635.20 | 0.82 | 17612.71 | 657.63 | 0.91 | 0.99 |
| PE(20:4_18:0) | 16751.17 | 401.89 | 14422.32 | 732.75 | 0.82 | 14081.70 | 704.88 | 0.91 | 0.99 |
| PE(18:0_22:4) | 4910.93 | 159.75 | 4295.92 | 189.21 | 0.83 | 4343.37 | 237.18 | 0.91 | 0.99 |
| PE(20:5_18:1) | 23866.08 | 888.78 | 19701.89 | 859.86 | 0.82 | 22537.86 | 1223.51 | 0.98 | 0.89 |
| PE(22:6_18:0) | 44505.92 | 1061.66 | 35883.56 | 1187.36 | 0.55 | 36361.00 | 2339.98 | 0.91 | 0.99 |
| PE(22:6_18:1) | 8984.56 | 311.20 | 7577.67 | 370.01 | 0.82 | 8387.16 | 790.87 | 0.98 | 0.97 |
| PE(P-18:1_20:4) | 325542.50 | 2443.19 | 316803.33 | 2092.15 | 0.83 | 326240.00 | 3688.06 | 0.98 | 0.87 |
| PE(P-18:0_22:6) | 5543.13 | 205.52 | 4581.01 | 152.93 | 0.82 | 5141.67 | 423.58 | 0.97 | 0.96 |
| PG(20:3_22:6) | 3211.00 | 57.51 | 3571.26 | 32.45 | 0.65 | 3288.70 | 75.15 | 0.98 | 0.78 |
| SM(d18:0/12:0) | 3190.96 | 93.33 | 2615.04 | 103.07 | 0.75 | 2692.10 | 115.56 | 0.91 | 0.99 |
| SM(d18:0/16:0) | 6803.63 | 202.27 | 7642.20 | 515.41 | 0.90 | 7769.51 | 392.31 | 0.91 | 0.99 |
| SM(d18:0/18:0) | 13499.27 | 307.41 | 13536.24 | 462.10 | 0.99 | 13686.47 | 980.40 | 0.98 | 0.99 |
| SM(d18:1/14:0) | 207344.17 | 7588.52 | 133827.78 | 7229.35 | 0.35 | 147404.00 | 11209.89 | 0.91 | 0.97 |
| SM(d18:1/16:0) | 102105.58 | 2339.03 | 109221.22 | 3860.37 | 0.91 | 97091.14 | 4279.14 | 0.96 | 0.87 |
| SM(d18:1/18:0) | 177550.00 | 3809.69 | 169832.22 | 5413.16 | 0.92 | 166126.00 | 8474.72 | 0.94 | 0.99 |
| SM(d18:1/20:0) | 22573.42 | 384.80 | 22201.44 | 547.05 | 0.94 | 19713.57 | 637.86 | 0.91 | 0.86 |
| SM(d18:1/22:0) | 10277.75 | 309.77 | 10657.74 | 327.54 | 0.94 | 8751.53 | 275.23 | 0.91 | 0.75 |
| SM(d18:1/24:0) | 11523.61 | 450.08 | 10610.52 | 758.11 | 0.92 | 9035.49 | 654.46 | 0.91 | 0.95 |
| SM(d18:1/16:1) | 10679.39 | 276.00 | 12025.70 | 283.19 | 0.82 | 11024.99 | 672.06 | 0.98 | 0.95 |
| SM(d18:1/18:1) | 22512.08 | 621.29 | 21407.89 | 785.88 | 0.92 | 20395.14 | 1158.10 | 0.94 | 0.97 |
| SM(d18:1/22:1) | 8156.04 | 102.61 | 8501.03 | 119.70 | 0.87 | 7610.54 | 285.15 | 0.91 | 0.82 |
| SM(d18:1/24:1) | 19609.82 | 664.82 | 19478.67 | 849.48 | 0.99 | 19851.49 | 1093.72 | 0.98 | 0.99 |
| SM(d18:2/24:1) | 11494.91 | 356.00 | 10164.47 | 465.53 | 0.87 | 9170.24 | 461.53 | 0.91 | 0.95 |
| TG(10:0_12:0_14:0) | 44486.25 | 231.95 | 45528.89 | 359.21 | 0.82 | 44569.71 | 420.63 | 0.98 | 0.91 |
| TG(8:0_14:0_16:0) | 21374.92 | 213.78 | 21741.11 | 284.40 | 0.92 | 20135.86 | 492.67 | 0.91 | 0.86 |
| TG(10:0_16:0_12:0) | 281261.67 | 1386.67 | 282193.33 | 1755.14 | 0.94 | 285050.00 | 674.94 | 0.94 | 0.97 |
| TG(8:0_16:0_16:0) | 22828.50 | 386.73 | 21296.22 | 364.57 | 0.82 | 23283.43 | 634.67 | 0.98 | 0.86 |
| TG(10:0_14:0_16:0) | 34804.00 | 278.37 | 33299.56 | 457.54 | 0.82 | 30459.71 | 471.95 | 0.39 | 0.75 |
| TG(12:0_14:0_16:0) | 16958.56 | 399.20 | 24115.44 | 654.91 | 0.09 | 14334.84 | 531.83 | 0.91 | 0.17 |
| TG(12:0_12:0_18:0) | 14009.57 | 306.95 | 16288.33 | 337.68 | 0.65 | 9406.56 | 446.75 | 0.39 | 0.17 |
| TG(14:0_14:0_16:0) | 26334.17 | 461.63 | 27214.56 | 478.02 | 0.92 | 18422.86 | 618.72 | 0.39 | 0.17 |
| TG(12:0_16:0_16:0) | 10589.93 | 221.22 | 13346.89 | 326.93 | 0.26 | 12928.54 | 431.28 | 0.91 | 0.97 |
| TG(14:0_16:0_16:0) | 47925.58 | 659.75 | 57245.89 | 1548.64 | 0.40 | 45406.43 | 1203.60 | 0.91 | 0.50 |
| TG(15:0_16:0_16:0) | 51665.83 | 849.67 | 58511.44 | 912.75 | 0.55 | 49465.29 | 1705.99 | 0.94 | 0.60 |
| TG(16:0_16:0_16:0) | 1219696.67 | 16149.09 | 1289466.67 | 16230.58 | 0.82 | 1360471.43 | 43504.79 | 0.91 | 0.91 |
| TG(14:0_16:0_18:0) | 30129.58 | 630.21 | 40262.89 | 1371.47 | 0.24 | 28730.29 | 824.09 | 0.94 | 0.46 |
| TG(16:0_16:0_17:0) | 31256.17 | 545.21 | 44784.11 | 1624.93 | 0.11 | 30745.43 | 573.12 | 0.98 | 0.43 |
| TG(16:0_16:0_18:0) | 2685091.67 | 39079.18 | 2932955.56 | 43978.51 | 0.75 | 3152657.14 | 101412.86 | 0.91 | 0.87 |
| TG(15:0_17:0_18:0) | 9614.67 | 133.47 | 10566.60 | 348.17 | 0.82 | 10151.24 | 250.56 | 0.91 | 0.97 |
| TG(16:0_17:0_18:0) | 16240.80 | 291.23 | 16636.44 | 439.60 | 0.94 | 15790.29 | 634.18 | 0.98 | 0.97 |
| TG(14:0_18:0_20:0) | 10640.35 | 179.66 | 11065.92 | 241.17 | 0.92 | 12445.24 | 534.31 | 0.91 | 0.87 |
| TG(16:0_18:0_18:0) | 3408925.00 | 47275.05 | 3623366.67 | 55998.36 | 0.82 | 3935871.43 | 117378.16 | 0.91 | 0.87 |
| TG(15:0_18:0_20:0) | 10025.28 | 156.30 | 10767.57 | 334.03 | 0.87 | 12754.14 | 364.12 | 0.39 | 0.77 |
| TG(17:0_18:0_18:0) | 89676.83 | 492.23 | 90669.33 | 517.43 | 0.92 | 94578.57 | 634.50 | 0.91 | 0.62 |
| TG(18:0_18:0_18:0) | 1503350.00 | 18461.88 | 1555855.56 | 24892.57 | 0.90 | 1681914.29 | 43496.16 | 0.91 | 0.87 |
| TG(16:0_18:0_20:0) | 18441.50 | 225.86 | 18917.22 | 266.35 | 0.92 | 21315.86 | 587.98 | 0.91 | 0.76 |
| TG(16:0_18:0_22:0) | 17386.08 | 224.55 | 17671.78 | 139.07 | 0.93 | 18607.14 | 418.51 | 0.91 | 0.87 |
| TG(14:0_16:0_16:1) | 31018.50 | 438.72 | 38394.00 | 674.39 | 0.09 | 32781.57 | 752.06 | 0.91 | 0.52 |
| TG(16:0_16:0_16:1) | 60517.75 | 1636.80 | 89475.00 | 3191.08 | 0.13 | 61285.00 | 2900.97 | 0.98 | 0.46 |
| TG(14:0_16:0_18:1) | 27116.92 | 983.92 | 45529.22 | 2459.14 | 0.20 | 30290.14 | 2441.31 | 0.94 | 0.75 |
| TG(16:0_16:0_18:1) | 83034.67 | 3335.54 | 184238.67 | 9680.97 | 0.04 | 92490.29 | 6765.18 | 0.94 | 0.03 |
| TG(16:0_18:0_18:1) | 84518.50 | 5589.71 | 285680.56 | 17935.88 | 0.03 | 119098.43 | 13155.95 | 0.91 | 0.04 |
| TG(17:0_18:0_18:1) | 10355.56 | 734.92 | 35201.57 | 2347.32 | 0.04 | 14686.26 | 1988.98 | 0.91 | 0.06 |
| TG(18:0_18:0_18:1) | 137680.25 | 10606.93 | 500598.56 | 32062.75 | 0.03 | 195714.71 | 21391.17 | 0.91 | 0.03 |
| TG(16:0_20:0_18:1) | 1567.23 | 92.42 | 4700.37 | 260.12 | 0.03 | 3253.93 | 259.45 | 0.03 | 0.78 |
| TG(16:0_16:1_16:1) | 23459.42 | 289.16 | 29437.22 | 765.68 | 0.15 | 24720.86 | 906.32 | 0.94 | 0.77 |
| TG(14:0_16:1_18:1) | 65753.67 | 690.38 | 63799.56 | 1177.76 | 0.92 | 68681.86 | 1375.44 | 0.91 | 0.87 |
| TG(16:0_16:1_18:1) | 50247.25 | 660.46 | 66209.67 | 1736.44 | 0.09 | 49594.57 | 1144.84 | 0.98 | 0.43 |
| TG(16:0_17:1_18:1) | 21405.83 | 798.54 | 38503.33 | 1767.86 | 0.09 | 20781.57 | 1435.80 | 0.98 | 0.43 |
| TG(16:0_18:1_18:1) | 168886.42 | 9223.97 | 478257.78 | 27653.65 | 0.03 | 215067.86 | 22159.66 | 0.91 | 0.04 |
| TG(16:0_18:0_18:2) | 18934.73 | 1235.20 | 60448.33 | 3748.56 | 0.03 | 23098.14 | 2371.25 | 0.94 | 0.02 |
| TG(16:0_16:1_20:1) | 102265.33 | 5099.04 | 278996.22 | 15560.52 | 0.03 | 129642.86 | 14201.03 | 0.91 | 0.04 |
| TG(18:0_18:1_18:1) | 51647.50 | 4447.71 | 206483.11 | 13420.10 | 0.03 | 80161.29 | 10617.10 | 0.91 | 0.04 |
| TG(14:1_16:1_18:1) | 33075.83 | 387.47 | 32751.44 | 742.48 | 0.94 | 29424.43 | 542.81 | 0.91 | 0.82 |
| TG(16:1_16:1_18:1) | 14559.68 | 297.50 | 17011.11 | 311.41 | 0.54 | 13737.51 | 539.49 | 0.94 | 0.50 |
| TG(16:0_16:1_18:2) | 33971.33 | 583.20 | 32155.11 | 925.79 | 0.90 | 25829.29 | 548.16 | 0.39 | 0.57 |
| TG(16:0_18:1_18:2) | 33784.25 | 748.59 | 59840.56 | 2367.02 | 0.03 | 44441.43 | 4395.79 | 0.91 | 0.82 |
| TG(18:1_18:1_18:1) | 92085.08 | 4469.32 | 225881.00 | 11826.99 | 0.03 | 125062.57 | 13235.18 | 0.91 | 0.50 |
| TG(18:0_18:1_18:2) | 17262.79 | 1268.37 | 48217.33 | 2639.53 | 0.04 | 21862.64 | 2920.99 | 0.94 | 0.05 |
| TG(16:1_18:1_18:2) | 19780.12 | 514.44 | 23352.78 | 566.15 | 0.66 | 23323.57 | 944.60 | 0.91 | 0.99 |
| TG(16:0_18:2_18:2) | 15898.68 | 344.22 | 21721.33 | 670.40 | 0.15 | 15835.36 | 957.62 | 0.99 | 0.57 |
| TG(18:1_18:1_18:2) | 20649.67 | 564.35 | 30000.89 | 806.10 | 0.09 | 24558.00 | 1928.95 | 0.91 | 0.86 |
| TG(18:1_18:2_18:2) | 17504.83 | 327.84 | 15919.78 | 422.81 | 0.82 | 17310.76 | 1180.72 | 0.98 | 0.97 |
| TG(16:0_18:2_20:4) | 16330.08 | 354.72 | 16090.00 | 211.35 | 0.94 | 14803.60 | 541.55 | 0.91 | 0.87 |

**Supplemental data 11.** lipid peroxidation after 6-OHDA-induced lesion formation

(A) Striatal sections showed Pex19 immunoreactivity in SN at 2, 3 and 5 weeks after unilateral lesion formation as assessed by immunofluorescence. (B) The graph shows the MDA content in CSF. Data are represented as mean ± S.E.M. *P < 0.05. Abbreviations: 6-OHDA, 6-hydroxydopamine; SN, substantia nigra; Pex19, peroxisomal biogenesis factor 19; MDA, malondialdehyde. Scale bar: 50 µm.


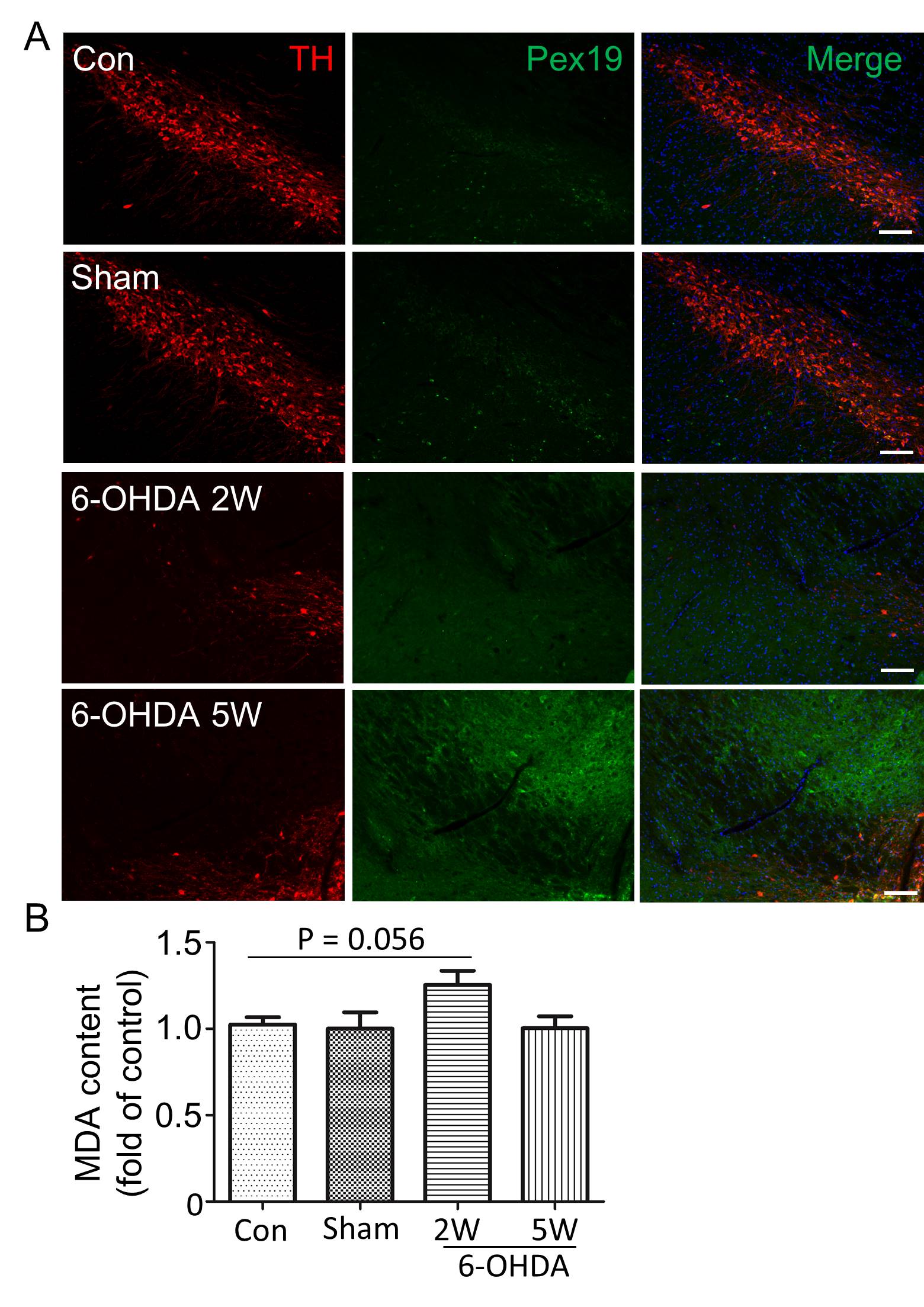

Supplement: Supplementary file 1 [file Data_Sheet_1.doc]
